# Belowground Microbiota and the Health of Tree Crops

**DOI:** 10.3389/fmicb.2018.01006

**Published:** 2018-06-05

**Authors:** Jesús Mercado-Blanco, Isabel Abrantes, Anna Barra Caracciolo, Annamaria Bevivino, Aurelio Ciancio, Paola Grenni, Katarzyna Hrynkiewicz, László Kredics, Diogo N. Proença

**Affiliations:** ^1^Department of Crop Protection, Agencia Estatal Consejo Superior de Investigaciones Científicas, Institute for Sustainable Agriculture, Córdoba, Spain; ^2^Department of Life Sciences, Centre for Functional Ecology, University of Coimbra, Coimbra, Portugal; ^3^Water Research Institute (CNR-IRSA), National Research Council, Rome, Italy; ^4^Department for Sustainability of Production and Territorial Systems, Italian National Agency for New Technologies, Energy and Sustainable Economic Development (ENEA), Rome, Italy; ^5^Institute for Sustainable Plant Protection, National Research Council, Bari, Italy; ^6^Department of Microbiology, Faculty of Biology and Environmental Protection, Nicolaus Copernicus University, Toruń, Poland; ^7^Department of Microbiology, Faculty of Science and Informatics, University of Szeged, Szeged, Hungary; ^8^Centre for Mechanical Engineering, Materials and Processes (CEMMPRE) and Department of Life Sciences, University of Coimbra, Coimbra, Portugal

**Keywords:** tree crops, belowground microbiota, biological control agents, endophytes, mycorrhiza, phytoparasitic nematodes, plant-growth-promoting microorganisms, soil-borne pathogens

## Abstract

Trees are crucial for sustaining life on our planet. Forests and land devoted to tree crops do not only supply essential edible products to humans and animals, but also additional goods such as paper or wood. They also prevent soil erosion, support microbial, animal, and plant biodiversity, play key roles in nutrient and water cycling processes, and mitigate the effects of climate change acting as carbon dioxide sinks. Hence, the health of forests and tree cropping systems is of particular significance. In particular, soil/rhizosphere/root-associated microbial communities (known as microbiota) are decisive to sustain the fitness, development, and productivity of trees. These benefits rely on processes aiming to enhance nutrient assimilation efficiency (plant growth promotion) and/or to protect against a number of (a)biotic constraints. Moreover, specific members of the microbial communities associated with perennial tree crops interact with soil invertebrate food webs, underpinning many density regulation mechanisms. This review discusses belowground microbiota interactions influencing the growth of tree crops. The study of tree-(micro)organism interactions taking place at the belowground level is crucial to understand how they contribute to processes like carbon sequestration, regulation of ecosystem functioning, and nutrient cycling. A comprehensive understanding of the relationship between roots and their associate microbiota can also facilitate the design of novel sustainable approaches for the benefit of these relevant agro-ecosystems. Here, we summarize the methodological approaches to unravel the composition and function of belowground microbiota, the factors influencing their interaction with tree crops, their benefits and harms, with a focus on representative examples of Biological Control Agents (BCA) used against relevant biotic constraints of tree crops. Finally, we add some concluding remarks and suggest future perspectives concerning the microbiota-assisted management strategies to sustain tree crops.

## Introduction

**Figure 1:**
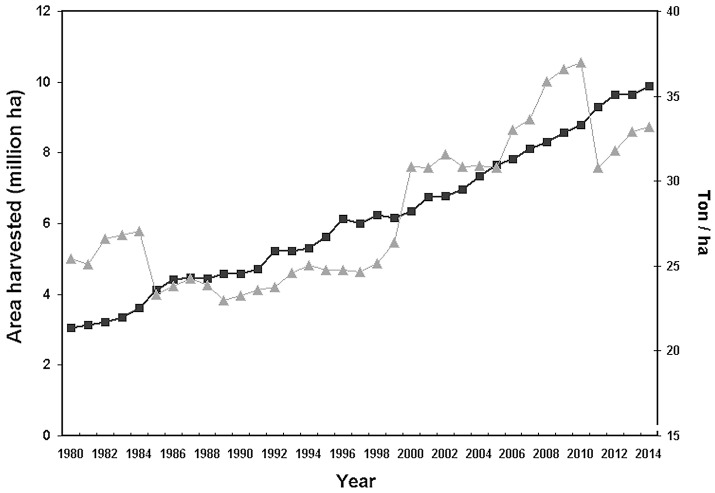
Total world surface (triangles) and yield/hectar (solid squares) of main tree crops (citrus, fresh and tropical, pome and stone fruits) (source FAOSTAT: *http://fenix.fao.org/faostat/beta/en/)*.

**Figure 2:**
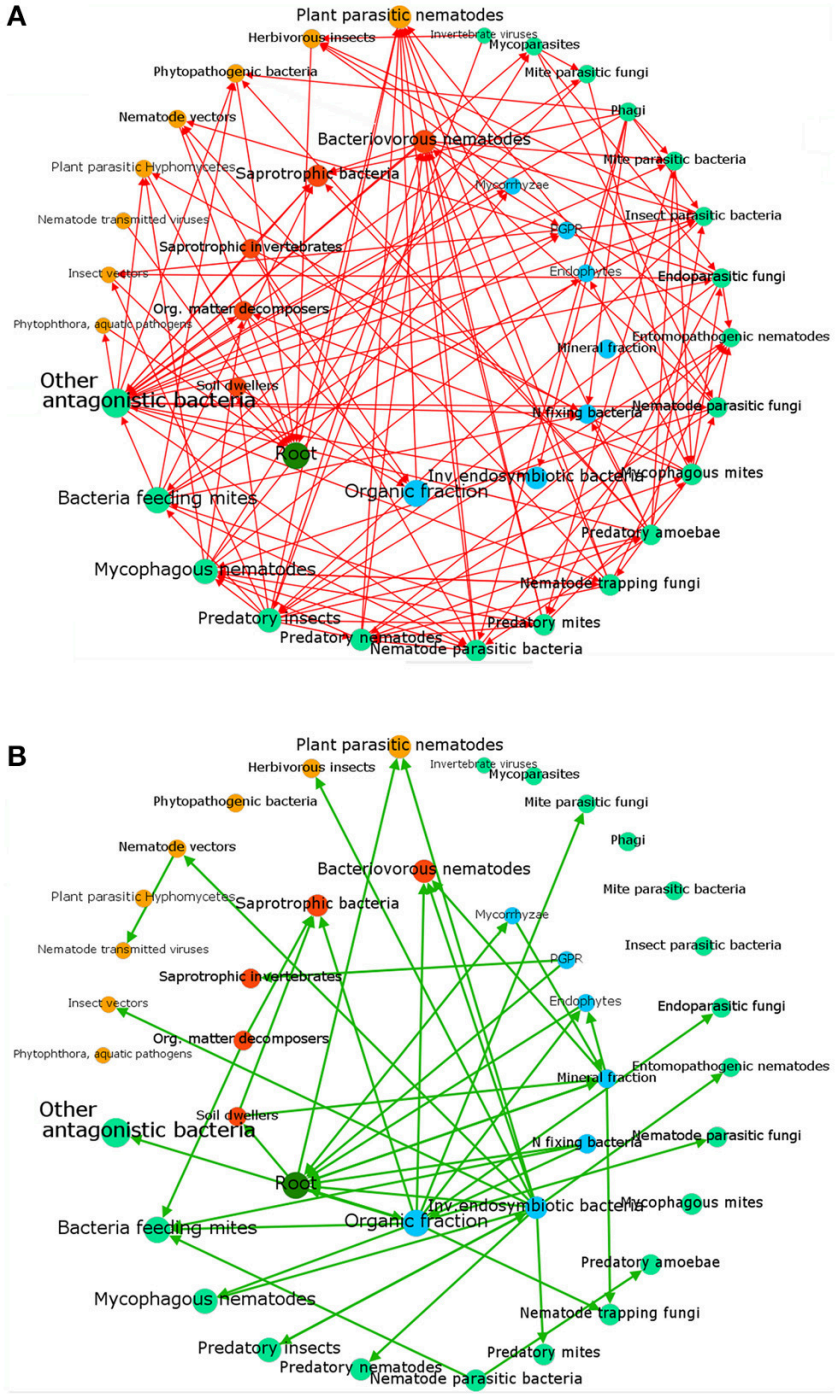
A simplified food web describing main soil components and their relationships. The nodes are classified by roles as: primary root (dark green), beneficial soil components, organisms or promoters, including soil factors (blue), decomposers (brown), pathogens (orange) and biocontrol agents or antagonists (pale green). Arrows show negative effects **(A)**, such as predation, parasitism, pathogenicity or **(B)** positive links, such as growth promotion, symbiosis or alimentary provision. Indirect factors such as those related to abundance, competition or other density-dependent effects are not included. Node labels and sizes are proportional to their connection level (number of edges). Analysis produced with Gephi (Bastian et al., 2009).

Tree crops are fundamental for human nutrition and warrant food security and stability of many farms. The surface covered by tree crops showed a growing trend in the last decade, approaching to a global acreage of 10 Mha for main fruit types with an ~20% increase in productivity during the period 2004–2014 (FAOSTAT, *http://fenix.fao.org/faostat/beta/en/)* (Figure 1). Plants (like trees) as well as the environment (such as soil) consist of complex and diverse assemblage of myriads of microbial species closely associated with their host, either as epiphytes or as endophytes (Trivedi et al., 2016). The association established by a plant and its microbiota (Lederberg, 2006) can be either stable, transient or fluctuating, enduring along the host lifetime determines its development, fitness, and health (Kowalski et al., 2015). The belowground microbiota is mostly comprised of bacteria and fungi belonging to the second trophic level (i.e., decomposers, mutualists, pathogens, parasites, and root-feeders) of the soil food web (Ingham, 1999) (Figure 2). Because of their size, nematodes per definition are not part of the soil microbiota, although they can play important roles in shaping its structure, including not only species belonging to the second trophic level (root-feeder nematodes) but also those ones of the third level (i.e., shredders, predators, grazers), particularly nematodes feeding on fungi and bacteria. Despite their parasitic behavior, phytoparasitic nematodes spend a considerable part of their life-cycle in the soil and represent the first group of plant parasites present in the soil. Therefore, the fraction of microorganisms linked to them can be considered as a specific compnent of the plant-associated microbiota (Vandekerckhove et al., 2000; Haegeman et al., 2009).

The study of the belowground microbiota has gained attention during the last years. Many studies have investigated soil belowground microbiota focusing on key issues such as the composition, structure, and functioning of these microbial communities and how they are built up and influenced by a range of factors [e.g., changing environment, varying weather/climatic conditions, (diffuse) pollution, anthropogenic actions, plant genotype, plant signals, etc.] [see, for instance (Doornbos et al., 2012; Bakker et al., 2013; Bulgarelli et al., 2013; Mendes et al., 2013; Lakshmanan et al., 2014; Fierer, 2017)]. Structural and functional modifications in the soil/rhizosphere microbiota have a crucial impact on aboveground ecosystems. In the particular case of trees, the trophic interactions established between the host and its associated belowground microbiota could be assumed, at least *a priori*, as more durable than that occurring in short-living, herbaceous species. Indeed, due to their perennial, long-living nature, it could be envisaged that belowground microbial communities associated with tree crops may be shaped by more persistent changes than those taking place in annual crops. Trees provide, in a more long-lasting way, an energy flow through photosynthesis, mobilizing nutrients as part of a continuous process leading to their recycling via the organic matter accumulation and its eventual decay. Moreover, due to the absence of annual rotation and lack of soil tillage, perennial tree crops also represent a stable food source not only for building up consortia of beneficial microbial communities but also for many root pathogens or parasites. Direct effects, due to deposition of organic matter and nutrients, could be more constant while indirect effects through agricultural inputs (i.e., application of fertilizers, pesticides, etc., irrigation and soil labor) would potentially work in a similar way as in annual crops. Being present on a time scale of years, and having a persistent, deeper root system, the impacts of tree crops (e.g., on nutrients mobilization, organic matter accumulation, parasites, etc.) largely differ from annual crops and thus cannot be considered as comparable. This is well illustrated by the currently-available and powerful metagenomic approaches (Colagiero et al., 2017). Overall, the events taking place between a tree crop and its associated whole soil microbiota have not been widely investigated.

In this study, we consider a tree crop as a woody, perennial plant with a distinct trunk, such as fruit, nut, and timber trees of economic importance, grown in orchards or in planted forests. Therefore, we exclude from this definition any palm “tree” species (*Arecaceae* family) as well as any other herbaceous perennial monocots (e.g., *Musa* spp., *Dracaena* spp., *Poaceae* family representatives, etc.) showing arborescent growth, since from both botanical and anatomical point of view they are not true trees. Tree crop ecosystems are of immense importance since they provide a range of products and ecosystem services. An increased understanding of the links between soil microbiota and trees is certainly helpful for the development of more effective and sustainable tree crop management strategies. Here, we (i) summarize methodological approaches used to unravel belowground microbial communities, with emphasis on tree crops; (ii) review the composition, distribution, and multitrophic networks of soil and root-associated microbiota, including endophytes, and the way they influence aboveground ecosystems in tree crops; (iii) examine the benefits (productivity, development, health and fitness, stress alleviation) and harms (mainly biotic stresses) for tree crops and woody plantations upon interaction with indigenous and introduced soil-borne (micro)organisms; and (iv) recapitulate strategies implemented for tree crop growth promotion.

## Methodological approaches to unravel the composition and function of belowground microbiota

Methods to assess the diversity, structure, and function of microbial communities can be categorized into three main groups, namely conventional, biochemical and molecular. Here, we summarize the advantages and limitations of main methodological approaches to study the composition and function of rhizosphere microbial communities, with emphasis on tree crops (Table 1).

**Table 1:** Methods to study belowground microbial communities.

**Methods**	**Advantages**	**Disadvantages**	**Crop examples**	**References**
**CONVENTIONAL AND BIOCHEMICAL**				
Growth on culture media, Enrichment cultures,	Microorganism isolation, pest control purposes, etc.; fast and low cost	Useful exclusively for cultivable microorganisms	Maritime pine (*Pinus pinaster*), *Pinus densiflora* and *P. thunbergii*	Islam and Ohga, 2013; Proença et al., 2017a
Enzymatic measurements	Functional activity; soil quality bioindicators; early indication of changes in soil; ecosystem disturbance estimate	Not necessarily linked to real ecological functioning and activity	Poplar (*Populus* sp.)	Winding et al., 2005
Community level physiological profiling (CLPP), sole-carbon-source utilization (e.g., API and BIOLOG)	Versatile for bacteria, fungi and specific carbon sources (BIOLOG); fast, highly reproducible and relatively inexpensive	It reflects potential metabolic diversity of fast growing microorganisms and not of overall *in situ* diversity; sensitive to inoculum density	Grapevine (*Vitis* spp.), olive, *Picea abies* forest	Söderberg et al., 2004; Montes-Borrego et al., 2013; Salomon et al., 2014; Lladó and Baldrian, 2017; Ruano-Rosa et al., 2017
Phospholipid fatty-acid analysis (PLFA)/Total Ester-linked Fatty Acid (ELFA)/Fatty acid methyl ester (FAME) analysis	Indicator of active microbial biomass opposed to non-living biomass; fingerprint of overall microbial communities (Bacteria, Archaea, Fungi)	Limited number of microbial groups identified	Diverse	Hill et al., 2000; Söderberg et al., 2004; Winding et al., 2005; Bastida et al., 2008; Francisco et al., 2016
**MOLECULAR**				
Fluorescent *In situ* Hybridization (FISH)	Direct identification and visualization of microbial groups based on the hybridization with a high number copies of their rRNA; it reflects microbial activity and makes it possible to quantify cell number; DNA extraction from soil is not required	Cells with low activity are not identified; it requires skill and experience in the case of low probe penetration and autofluorescence; information obtained is dependent on the availability of designed probe(s)	*Cactaceae*, rosemary (*Rosmarinus officinalis*)	Hill et al., 2000; Barra Caracciolo et al., 2005, 2015; Lopez et al., 2011
Total microbial abundance (DAPI counts); cell viability	Detection of all microbial cells independently from their physiological state	Overestimation of cell number if they are just dead but with an intact DNA	Poplar, rosemary	Barra Caracciolo et al., 2015; Ancona et al., 2017
Genetic fingerprinting techniques (DGGE/TGGE, T-RFLP)	Sensitive to variation in DNA sequences; bands can be excised, cloned and sequenced for subsequent identification	Multiple bands for a single species can be generated due to micro-heterogeneity: can be used only for short fragments; complex communities may appear smeared due to a large number of bands; Inconsistencies (gel to gel variation)	*Citrus* spp., *Elsholtzia splendens* and *Commelina communis*, pine (*Pinus* sp.), cork tree (*Quercus suber*)	Hill et al., 2000; Araújo et al., 2002; Kuklinsky-Sobral et al., 2005; Winding et al., 2005; Singh et al., 2007; Sheng et al., 2008; Wang et al., 2008; Sun et al., 2010; Bevivino et al., 2014; Ng et al., 2014; Proença et al., 2017a
qPCR, RT-qPCR, dPCR	Quantitative and highly sensitive for species identification and functional genes; easy to implement and cheap; specific amplification confirmed by melting curve analysis	Can only be used for targeting of known DNA sequences; DNA impurities and artifacts may lead to false-positives or inhibit amplification	Poplar, switchgrass (*Panicum virgatum*)	Liang et al., 2014; Cai et al., 2015
Next-generation sequencing (NGS)	Rapid to assess biodiversity and abundance of many species/organizational taxonomic units simultaneously	Massive amount of sequencing data of DNA (genomic or PCR amplified fragments) or RNA error distribution within reads of a library; insertions or substitution errors; relatively expensive; replication and statistical analysis are essential; computational intensive; challenging in terms of data analysis	Maritime pine (*Pinus pinaster*)	Proença et al., 2017a
DNA sequence analysis of the internal transcribed spacer (ITS) region for mycorrhizal studies	Fast and accurate for the identification of mycorrhizal fungi and characterization of their distribution.	Relatively expensive, especially in case of metagenomic analyses	Ectomycorrhizas of poplar (*Populus nigra*×*maximowiczii*) and willow clone (*Salix viminalis*) cultivated as SRF, mycorrhizal fungi of willow (*Salix* spp. L.) from hydrocarbon-contaminated soils, AMF of *Acacia gerrardii* under salt stress	Hrynkiewicz et al., 2010; Hassan et al., 2014; Hashem et al., 2016;

*A few illustrative examples of herbaceous plants are also given*.

### Conventional and biochemical methods

Culture-based methods constitute a good complement to DNA-based approaches. However, they are extremely biased regarding the actual evaluation of microbial genetic diversity since only <1% of the total number of prokaryotic species present in the environment are culturable. Several improved procedures and media mimic natural environments in terms of nutrients, oxygen gradient, pH, etc. maximizing the cultivable fraction of soil-borne microbial communities (Gravel et al., 2007). In addition, the number of colony-forming units (CFU) is positively correlated with enzymes and respiratory activity. This approach may be applied to characterize the relative abundance of active microorganisms with certain functions or trophic requirements (Blagodatskaya and Kuzyakov, 2013). Even though culture-dependent methods are not ideal for evaluating the actual composition of natural microbial communities when used alone, they are useful for understanding growth habits, development, and potential functions of soil and rhizosphere microorganisms (VanInsberghe et al., 2013; Bevivino and Dalmastri, 2017).

Biochemical methods enable the assessment of soil microbiota activities of both the overall microbial community (e.g., dehydrogenase activity) and specific components (e.g., ammonia-oxidizing bacteria). The release of labile compounds, including enzymes, by living roots or lysis of root cells, stimulates microbial activity and growth in a similar way as rhizodeposits (Loeppmann et al., 2016). Consequently, localization of easily available C yields hotspots of microbial abundance and activities, frequently termed as the “rhizosphere effect” (Reinhold-Hurek et al., 2015; Thijs et al., 2016). Extracellular enzyme activities in the rhizosphere are higher compared to root-free soils, similarly to total microbial biomass and activity measured as respiration or growth rates (Allison and Vitousek, 2005; Ancona et al., 2017). Roots and associated mycorrhizal communities are known as major producers of β-glucosidases and acid phosphatases (Conn and Dighton, 2000). Despite soil enzymes being partly of plant origin, microorganisms constitute the main source of enzymes mediating the cycling of major nutrients (C, N, P, and S).

One approach to characterize the soil microbial communities is the Community Level Physiological Profiling (CLPP), in which species are identified based on utilization of different carbon sources with EcoPlateTM (Biolog, Inc.). CLPP yields information on both function and structure of part of a microbial community metabolically active under plate conditions (Garland and Mills, 1991). The BIOLOG® advantages include the identification of physiological profiles of a microbial community as a whole (Stefanowicz, 2006). However, most bacterial cells in natural ecosystems are inactive and the substrates available in BIOLOG® plates are not necessarily relevant from the ecological point of view, and do not reflect the diversity of substrates found in the environment (Konopka et al., 1998). This methodology has been applied to compare functional diversity of communities from rhizosphere and non-rhizosphere soils (Söderberg et al., 2004), from rhizospheres of different plant species (Grayston et al., 1998), and to link microbial functional diversity of olive rhizosphere soil to management systems in commercial orchards (Montes-Borrego et al., 2013). While limitations of this methodology for the characterization of whole communities are well known, it continues to be used in combination with molecular approaches to identify the copiotrophic, fast-growing fraction of the bacterial community of soil environments as those from coniferous forests, where oligotrophic taxa are usually dominant (Lladó and Baldrian, 2017).

Biochemical methods can also be used to assess microbial community structure and to perform a phenotypic fingerprinting of the main groups (Gram-positive and Gram-negative bacteria, fungi, etc.) in the rhizosphere. This is the case of the phospholipid-derived fatty-acid (PLFA) and the total ester-linked fatty-acid (ELFA) methods (Sharma and Buyer, 2015; Hinojosa et al., 2016). As the fatty-acid side chains are rather unique among the various life forms, these molecules are widely used as taxonomic and phylogenetic biomarkers to describe the structure and size of microbial communities in soil and rhizosphere samples (Debode et al., 2016; Francisco et al., 2016). Phospholipid fatty-acids are found exclusively in cell membranes and not in other parts of the cell as storage products. This is important as cell membranes are rapidly degraded and the component PLFA is quickly metabolized following cell death. Consequently, phospholipids can serve as important indicators of active microbial biomass as opposed to non-living biomass. These methods are useful for assessing the structure of soil microbial communities and for determining effects of soil disturbances such as cropping practices, pollution, and changes in soil quality. For example, PLFA analysis was successfully used to investigate the impact of *Populus* spp. grown as short rotation coppice (SRC) on the microbial communities of arable soils (Baum et al., 2013).

### Molecular methods

Molecular methods have provided a more-in-depth understanding of the occurrence and phylogenetic diversity of soil microbial communities (Tiedje et al., 1999; Fakruddin and Mannan, 2013). Polymerase chain reaction (PCR)-based approaches are commonly used for phylogenetic assignments. Small subunit rRNA genes (for instance, the 16S small subunit ribosomal RNA [16S rRNA] for prokaryotic cells) are amplified from soil-extracted nucleic acids. Microbial rRNA gene sequences can then be sequenced and identified using appropriate databases (e.g., NCBI GenBank, EMBL, EzBioCloud, etc.) and compared with those of known microorganisms (Janssen, 2006). Similarly, the identification of soil fungi and fungal symbionts associated with previously selected and characterized mycorrhizas is based on sequence analysis of gene fragments from the large-subunit rRNA (LSU) or their internal transcribed spacer (ITS) regions (Porras-Alfaro et al., 2014). Taxonomic and phylogenetic affiliation of fungi can be based on widely available databases like the NCBI GenBank or on the stable and reliable platform UNITE, designed for sequence-based identification of ectomycorrhizal asco- and basidiomycetes.

Molecular-based approaches have revealed an extraordinary taxonomical and functional diversity of microorganisms. To study the population structures and dynamics of microbial communities, genetic fingerprinting techniques such as Denaturing Gradient Gel Electrophoresis (DGGE) were developed (Muyzer et al., 1993). Nowadays, DGGE can be used as a first approach to visualize main differences in a given microbial community and subsequently high-throughput sequencing (HTS) can be applied to have a deeper understanding of the microbiota composition (Di Lenola et al., 2017; Proença et al., 2017a). This methodology has been implemented in different fields and it is very common in soil microbiology studies (Bevivino et al., 2014; Ng et al., 2014), or to assess the aboveground microbial structure of trees (e.g., maritime pine, *Pinus pinaster* Ait.) (Proença et al., 2017a). Other community profiling techniques include temperature gradient gel electrophoresis (TGGE), single-strand conformation polymorphism (SSCP), terminal restriction fragment length polymorphism (T-RFLP), amplified rDNA restriction analysis (ARDRA), and amplified ribosomal intergenic spacer analysis (ARISA) (Anderson and Domsch, 1989; Anderson and Cairney, 2004). These methods can also provide detailed information about community structure in terms of richness, evenness and composition and permit to identify selected species and functional genes involved in specific processes. Nevertheless, these qualitative PCR-based methods do not provide information on the gene copy numbers. To achieve that, implementation of qPCR (quantitative PCR) is needed whereas RT-qPCR (reverse transcription qPCR) is informative about the expression of a specific gene (Stella, 2014). However, the phylogenetic characterization of prokaryotic cells based on DNA extraction from soil does not reflect the activity of rhizosphere microbial community, as DNA may also proceed from dead or inactive cells. Likewise, the analysis of biodiversity based on the molecular identification of single ectomycorrhizal roots or arbuscular spores, and the application of cloning for identification of arbuscular mycorrhizal fungi (AMF), have some limitations difficulting a reliable portrait of the microcosm environment condition. Thus, a novel sequence-based method was developed to describe AMF communities, coupling the previously established AMF-specific PCR primers that amplify a *c*. 1.5-kb long and AMF-specific pSSU-ITS-pLSU fragment with single molecule real-time (SMRT) sequencing (Schlaeppi et al., 2016). Finally, substantial progress has been also made to facilitate the quantitative detection of individual nematode taxa on the basis of small subunit ribosomal DNA-based (SSU-rDNA) monitoring of nematode assemblages (Vervoort et al., 2012). In complex environments, such as soil, the newly developed digital polymerase chain reaction (dPCR) has been recently applied to quantify the absolute concentration of DNA targets or functional genes in soil (Kim et al., 2014; Cavé et al., 2016). This technology represents a promising tool enabling to examine the dynamics of soil microorganisms and to target pathogen-derived nucleic acids in environmental samples (Farkas et al., 2017).

### Epifluorescence microscope-based methods

Epifluorescence microscope-based methods do not need DNA extraction from soil, enabling direct visualization of microbial cells/structures under an epifluorescence microscope. The total direct count, cell viability (live/dead) and Fluorescence *In situ* Hybridization (FISH) are reliable and commonly used methods. The total direct count allows assessing microbial abundance through a DNA fluorescent intercalant such as DAPI, which can detect all microbial cells in a rhizosphere sample regardless of their physiological state and metabolic activity (Lew et al., 2010; Barra Caracciolo et al., 2015). Similarly, two fluorescent dyes, SYBR™ Green II and propidium iodide, can be used to discriminate between viable and dead cells (Ancona et al., 2017). Finally, FISH enables phylogenetic *in situ* identification and quantification of soil and rhizosphere communities at different phylogenetic levels (from domain to species), by using fluorescent labeled rRNA-targeted oligonucleotide probes in single cells. rRNA-targeted probes that occur in a large copy number detect specific sequences of rRNA in single cells. Since only viable and active cells possess a sufficient number of undamaged ribosomes, they act as indicators of the physiological state of cells (Di Lenola et al., 2017). The detection of FISH-stained cells can be hampered by strong soil background autofluorescence which is avoided by applying a density gradient centrifugation to extract the detachable bacteria from soil particles (Barra Caracciolo et al., 2005, 2010). FISH has been successfully applied in analyses of active microorganisms in the rhizosphere (Barra Caracciolo et al., 2015) including endophytes (Kutter et al., 2006; Lopez et al., 2011). The main limitations of this method are: (i) its inability to detect unknown species and those with low ability, or for which specific probes have not been designed yet, and (ii) probe's difficulty to enter into Gram-positive cells under specific conditions.

### Meta-omic approaches

The recent development of HTS-based metagenomic analyses has further contributed to unveil either microbial or plant functioning in the rhizosphere, to yield a global view of the structure and diversity of the rhizosphere microbiota (Leveau, 2007; Barberán et al., 2012; Lindahl et al., 2013; Mendes et al., 2013; Hassan et al., 2014). The implementation of genomic methods to microbial assemblages is commonly used to describe communities overcoming biases inherent to PCR amplification of a single gene. The classical metagenomic strategy, as defined by Handelsman and colleagues (Handelsman et al., 1998), involves the following steps: DNA isolation, fragmentation and cloning, library screening, sequencing of interesting clones, and DNA comparison. Actually, three major and often overlapping directions can be recognized: the first trend aims at linking phylogeny to function; the second involves the discovery of genes or functions of interest; and the third is the mass sequencing of environmental samples which offers a more global (or systems-biology) view of the community under study (Steward Rappé and Rappé, 2007).

The HTS or next-generation sequencing (NGS) technology is experiencing a rapid development, providing wide and in-depth views in metagenomics. Several protocols and tools, including bioinformatic resources, are available for these studies. A number of HTS platforms have been developed and are widely used, including the Illumina (e.g., HiSeq, MiSeq), Roche 454 GS FLX+, SOLiD 5500 series, and Ion Torrent/Ion Proton platforms. Currently, the majority of microbial ecology studies implement HTS by focusing on either targeted gene sequencing with phylogenetic or functional gene targets or on shotgun metagenome sequencing (Pervaiz et al., 2017).

Most of the bacterial community studies have depended on a single gene, such as the hypervariable regions of the 16S rRNA gene, to assess taxonomic diversity and to determine which bacteria are present in a community. Other useful targets for bacterial community studies based on single amplicon sequencing include the type I chaperonins (*cpn60* gene) (Links et al., 2012). However, these “metabarcoding” methods (*sensu stricto* they cannot be considered as metagenomic approaches since they are just based on libraries of single amplicons) are limited by short read lengths, sequencing errors, differences arising from the different regions chosen, and difficulties in assessing operational taxonomic units (OTU). Shotgun metagenomics sequencing avoids many of the biases encountered in amplicon sequencing because it does not require amplification prior to sequencing (Fierer et al., 2012; Sharpton, 2014). Application of metagenomic analysis also paves the way for scientists to build fundamental knowledge on fungal communities in the environment. Actually, the metagenomics assessment of fungal diversity is common not only for soil but also for plant samples (mycorrhiza, endophytes), enabling detailed determination of all fungal trophic groups: saprophytic, pathogenic, endophytic, and symbiotic (Lindahl et al., 2013).

Further technologies such as the nanopore sequencing (with mini flow cells such as the MinIon™ by Oxford Nanopore™), or the PacBio™ sequencing based on ionic readings are gaining popularity due to their capability to sequence very long reads (up to several kilobases) in milliseconds and without amplification (Branton et al., 2008; Singer et al., 2016). Some of these novel approaches are promising, since they combine easy use and/or portability with a massive data production. They have the potential to sequence all the retrotranscribed rDNA molecules present in a sample, thus accounting for a direct identification of active species. In the light of experimental assays applied to plants, the information that may be gained through these studies are higher than the limits considered a few years ago, and often exceed the analytical potential of the bioinformatic resources eventually applied.

By using the above methodological approaches, the diversity, structure, and functioning of fungal and bacterial communities, endophytic and/or rhizospheric, were studied in tree species including *Populus deltodies* (Gottel et al., 2011; Shakya et al., 2013), native forest species (Buée et al., 2009), and conifers (Baldrian et al., 2012; Proença et al., 2017a). For instance, these studies were instrumental to link the so-called core (bacterial) microbiota to specific ecological niches in a given species and, more importantly, under field-grown conditions (Beckers et al., 2017). Based on sequencing data it is also possible to predict the function of a microbial community by using the bioinformatic tools PICRUSt (Langille et al., 2013) and tax4fun (Aßhauer et al., 2015).

Metatranscriptomics, in which total environment RNA is sequenced, is applied to reveal and compare active community members and metabolic pathways (Urich et al., 2008; Turner et al., 2013). Although the analysis of total rRNA has been widely used to profile microbial communities in soil (Carvalhais et al., 2012), the gene expression of microbes in the rhizosphere is much less studied due to the difficulty to obtain sufficient material under controlled conditions from a highly variable and irregular niche. Nevertheless, metatranscriptomics has been used to identify genes expressed by eukaryotes in forest soils, to study the fungal and bacterial responses to N deposition in two forests dominated by sugar maple (*Acer saccharum* Marsh), or to analyse ectomycorrhizal roots and the genes active in the *Piloderma*–*Pinus* symbiosis (Damon et al., 2012; Liao et al., 2014; Hesse et al., 2015). Finally, the sensitivity of current metabolomic platforms represents an important constraint showing that this approach cannot solve all rhizosphere-signaling relations such as chemical communications and interactions (van Dam and Bouwmeester, 2016).

## Factors influencing belowground microbiota associated with tree crops

A long-living host may establish a durable interaction with its associated microbiota compared to that taking place in annual and/or herbaceous plants. Nevertheless, the composition and structure of the associated microbiota in any given tree crop undergo alterations along time and space due to factors such as environmental (sudden/long-term) changes, physical-chemical soil properties, anthropogenic actions, agronomical practices, climatic factors, plant developmental stage, (a) biotic stresses, etc. Depending on the tree crop under study, this range of factors may have either major or minor influence on the entire belowground microbial communities or on some of their specific components (Caliz et al., 2015).

Temperature and precipitation along with seasonal variations are among the main climatic/weather components controlling microbial growth and reproduction; therefore, these abiotic factors may substantially influence the soil microbiota of tree crop plantations and forests. Okada and colleagues found that autumn precipitation in the preceding year was a crucial factor influencing the biomass of ectomycorrhizal fungi (EMF) in a 40/50-year-old *Pinus densiflora* L. forest, while soil water availability for EMF and host plant roots in the growing season could positively impact ectomycorrhizal biomass in subsequent seasons (Okada et al., 2011). With the aim of simulating realistic future drought conditions, Felsmann and colleagues studied the effects of reduced precipitation for one growing season on the bacterial community of beech (*Fagus sylvatica* L.) and conifer forests (Felsmann et al., 2015). They found that moderate drought induced by the precipitation manipulation treatment significantly affected the active but not the total bacterial community, proposing that there is an adequate resistance of the soil microbial system over one growing season. In soils of a temperate beech forest, seasonality, resource availability and climatic factors (temperature and moisture) affected the community structure and abundance of Archaea and Acidobacteria indicating the high metabolic versatility and adaptability of these prokaryotic groups to environmental changes (Rasche et al., 2011). Finally, the effects of annual and interannual environmental variability of temperature, precipitation and chemical resources on soil fungi associated with an old-growth, temperate hardwood forest were investigated (Burke, 2015). Fungal communities were found to significantly vary by the season, sampling location, and depth with differences being consistent between years. Fungal species within the community were not consistent in their seasonality or preference for certain soil depths, but some of them were found to be consistently correlated with soil chemistry across the sampled years.

The soil properties are modified by a range of processes occurring during tree growth, which in turn affect rhizosphere microbial communities. Plant roots can influence the surrounding soil and inhabiting organisms (Lakshmanan et al., 2014). Roots release low-molecular-mass compounds (e.g., sugars, amino acids and organic acids), polymerized sugar, root border cells, and dead root cap cells. These rhizodeposits are used as carbon sources by soil microorganisms and can also contain secondary metabolites, such as antimicrobial compounds, nematicides, and flavonoids that are involved in establishing symbiosis or in warding off pathogens and pests, thereby acting as a crucial driving force for multitrophic interactions in the rhizosphere (Bais et al., 2006; Oldroyd, 2013). Experimental data from citrus crops parasitized by the insect pest *Diaprepes abbreviatus* in Florida showed that roots release specific volatile organic compounds (VOC) that attract entomopathogenic nematodes (EPN), with beneficial effects observable on the pest regulation. Also, plant-parasitic nematodes (PPN) revealed a positive tropism toward parasitized roots, mediated by one or more of the VOC components (Ali et al., 2010, 2011, 2013). This effect may be also significant for the microbiota associated with these nematode groups because several microbial species with a beneficial impact are passively dispersed by EPN and PPN. Soil pH, another important driver of soil microbial communities, can locally increase or decrease by up to two units in the rhizosphere due to the release and uptake of ions by roots (Hinsinger et al., 2009). Water uptake and root respiration affect soil oxygen pressure, thereby influencing microbial respiration. Soil nutrient availability can be modified in the rhizosphere by plant uptake and by the secretion of chelators, such as phytosiderophores, to sequester metallic micronutrients (Philippot et al., 2013).

The host plant can be considered as the primary biotic factor influencing the composition of soil microbiota associated with tree crops. The plant cover and crop types have an impact on the belowground microbial diversity, as shown by studies on soil metagenomes (Uroz et al., 2016; Colagiero et al., 2017). Structure and composition of fungal and archaeal communities proved to be dependent on the tree species, while bacterial communities differed between bulk soil and the rhizosphere but not between host trees. Similar results were obtained by Urbanová and collegues who demonstrated that fungal communities were strongly related to tree species while bacterial communities rather to root exudates (Urbanová et al., 2015). The composition of the nematode community in the rhizosphere soil is also influenced by the host genotype, as revealed by studies performed in olive (Palomares-Rius et al., 2012). Nematodes are also among the biotic factors influencing the composition of soil microbiota associated to tree crops, as shown by the differences induced on the AMF communities colonizing galls and roots of peach, *Prunus persica* (L.) Batsch, infected by the root-knot nematode *Meloidogyne incognita* (del Mar Alguacil et al., 2011).

Regarding anthropogenic factors, pollution caused by industrial and mining activities can shape microbiota associated with tree crops and timber trees. The effects of long-term metal pollution on soil microbial communities were evaluated along two soil gradients of forests with Scots pine, *P. sylvestris* L., and common beech as the dominant tree species (Azarbad et al., 2015). Metal pollution significantly affected bacterial community structure causing changes in the relative abundance of specific bacterial taxa resilient to metal pollution and increased frequency of certain metal-resistance genes, suggesting a link between microbial community composition and their functional potential in long-term polluted forest soils. The activity of timber harvesting was also shown to exert a significant and persistent effect on soil bacterial and fungal communities in Northern coniferous forests via organic material removal and soil compaction (Hartmann et al., 2012). Among the components of microbiota, plant symbionts like EMF and saprobic taxa of bacteria and fungi were the most sensitive to harvesting disturbances. The diversity and structure of soil bacterial and fungal communities remained significantly altered by harvesting disturbances, even more than a decade after harvesting. A subsequent study (Hartmann et al., 2014) revealed that physical soil disturbance during logging-associated compaction induced profound and long-lasting changes in the forest soil microbiota and associated soil functions, significantly reducing bacterial and fungal abundance, increasing alpha diversity and persistently altering the microbiota composition with a maximum impact observed 6–12 months after compaction. Fungi were less resistant and resilient than bacteria, with ectomycorrhizal species detrimentally affected by compaction, while saprobic and parasitic fungi were proportionally increased. Bacteria capable of anaerobic respiration, including metal, sulfur, and sulfate reducers from Proteobacteria and Firmicutes, were found to be significantly associated with compacted soils. Agronomical management systems also greatly influence the structure and functioning of soil microbial communities associated with tree crops. For instance, Montes-Borrego et al. (2013) revealed in a comparative analysis of organic and conventional olive farming systems in southern Spain, how management practices affected the chemical and biological soil properties indicating that olive orchards under organic management exhibited higher microbial diversity compared to conventionally managed orchards. The structure and diversity of phytoparasitic nematode communities infesting olive orchards are also, but not exclusively, influenced by soil management practices (Palomares-Rius et al., 2015). Indeed, this study concluded that soil physicochemical factors such as texture, pH, and extractable K, the climatic parameters minimum and maximum temperatures, and olive cultivar as the key agronomic variable were factors driving the population levels and community structure of olive phytoparasitic nematodes. An advanced citrus production system with daily fertigation rates have been applied in Florida to contrast the bacterial disease huanglongbing, by shortening the trees production cycle. This system increased the densities of some microbial antagonists of PPN such as *Catenaria* or other parasitic fungi, associated to a higher root biomass. However, some effects were also found on the densities of EPN, which showed opposite responses for steinernematid or heterorhabditid species (Campos-Herrera et al., 2014).

## Belowground microbiota and tree crops: benefits and harms

Beneficial soil/root microbiota can promote plant growth directly (i.e., biofertilization, phytostimulation) and/or indirectly (i.e., suppressing plant diseases and pests). Alleviation of stress due to environmental pollutants or heavy metals [i.e., (phyto)rhizoremediation)], drought or salinated soils, are mediated by the activity of the plant-associated microbiota. Trophic interactions established between the host plants and their associated microbiota at the root level provoke effects influencing aboveground ecosystems. Moreover, long-term associations (i.e., nodule-forming bacteria able to fix N_2_, ecto- and endomycorrhizal symbioses, non-symbiotic plant-growth-promoting rhizobacteria [PGPR] and fungi [PGPF], endophytes, etc.) may influence aboveground ecosystems in ways other than direct plant growth promotion. Successful associations should be based on the capacity of the microbes to modulate the plant host immunity. The dialogue established between plants and (components of) their microbiota are likely variations of a common theme where the boundaries among symbiotic, pathogenic or endophytic associations are, indeed, fuzzy (Zamioudis and Pieterse, 2012; Mercado-Blanco and Lugtenberg, 2014). Responses triggered in the plant as a consequence of the interactions taking place at the root level have an effect on aerial parts. Induction of systemic defense responses is a clear example that may affect plant health by triggering an enhanced resistance status against a range of phytopathogens and/or pests (Pieterse et al., 2014). The challenge is to understand these responses and how they disturb aboveground ecosystems, individuals or specific plant organs.

### Benefits

#### Mycorrhiza

**Figure 3:**
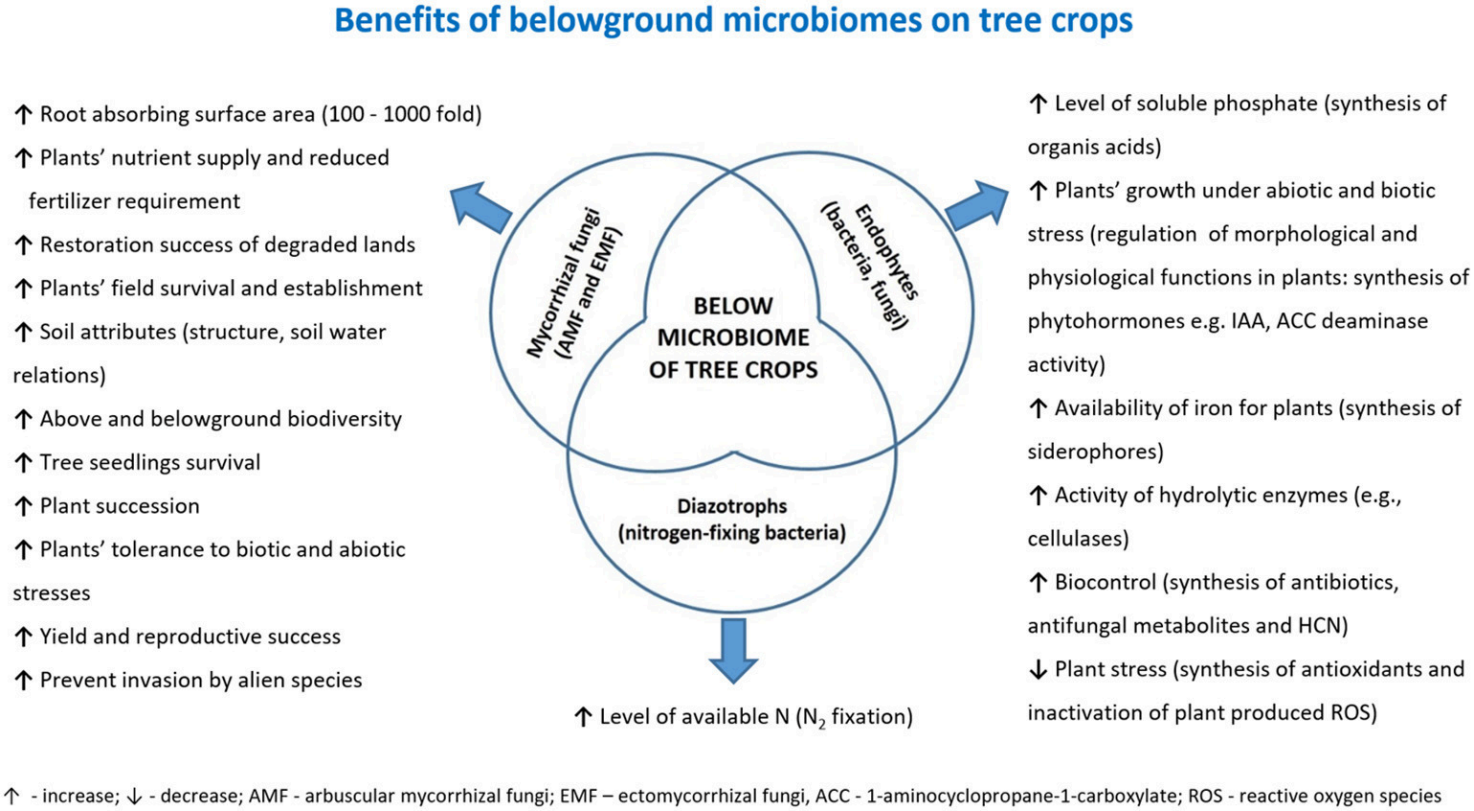
Summary of the benefits that belowground microbiota (or some of their components) may confer to tree crops.

Most of the known tree crops, i.e., fruit trees cultivated in orchards (e.g., olive, apple, *Malus domestica* L., pear, *Pyrus* sp., cherry, *Prunus* sp., plum, *P. domestica* L., peach, apricot, *P. armeniaca* L., etc.) or fast growing tree species cultivated in SRF systems for biomass production (e.g., willow, poplar, alder, *Alnus* sp., ash, *Fraxinus* sp., birch, *Betula* sp., eucalyptus, *Eucalyptus* sp., etc.) form stable symbioses with mycorrhizal fungi. Tree crops can form two types of mycorrhizas differing in morphology: ectomycorrhizas (EM) or arbuscular mycorrhizas (AM). Moreover, some tree crops can form dual EM/AM (e.g., willow, poplar), although a trend toward greater fractional colonization with EM and lower colonization with vesicular-arbuscular mycorrhiza (VAM) has been observed (Moyersoen and Fitter, 1999). Mycorrhizal fungi promote plant growth, aid nutrient uptake (reduced fertilizer requirement), increase yield, reproductive success and tolerance to abiotic (e.g., pollution, drought, salinity) and biotic (pathogens, herbivores, low microbial diversity in the soil) stresses, thereby improving field survival and establishment (Allen, 2006; Hrynkiewicz and Baum, 2012; Al-Karaki, 2013; Khabou et al., 2014; Manaut et al., 2015). Therefore, tree crops with well-established mycorrhizal symbiosis are characterized by increased adaptation level to edaphic parameters observed under unfavorable soil conditions. Direct and indirect beneficial effects of mycorrhizal fungi on plant growth and development are summarized in Figure 3.

Noteworthy, positive effects of mycorrhizal fungi on fruit tree growth can be detected only a few years after planting. Indeed, during the first year of a tree growing in an orchard, it may happen that mycorrhizal fungi use some nutrients that could nourish the tree's own growth (Borkowska, 2002). In the case of ectomycorrhiza associated to *Salix viminalis*, a stronger growth of the plant can be already observed three months after EMF occurrence (Hrynkiewicz et al., 2012). Beneficial effects of mycorrhizal symbiosis may vary considerably between fungal and plant species, and with environmental conditions (e.g., physical-chemical soil parameters, climate, etc.).

Mycorrhizal associations of fast-growing trees play also a key role in host tolerance to unfavorable soil conditions, increasing phytoremediation efficiency of heavy metals and organic contaminants (Vervaeke et al., 2003; Baum et al., 2006). The most numerous group of EMF symbionts, along with the highest level of EMF colonization, observed in natural stands of tree crops, belong to orders Thelephorales (*Tomentella* sp.), Pezizales (*Tuber* sp., *Geopora* sp.) and Agaricales (e.g., *Hebeloma* sp., *Cortinarius* sp.). The mechanisms of action responsible for tolerance of EMF to adverse environmental conditions are not yet fully understood. Some results suggest that melanin or thelephoric acid present in the fungal mycelium can act as a protective interface between fungal metabolism and (a)biotic environmental stressors. Species of *Geopora* have been found to be the principal EMF symbionts of willows planted for restoration in fly ash, with high potential to survive under harsh environmental conditions (Hrynkiewicz et al., 2009; Gehring et al., 2014). Ectomycorrhizal associations, dominated by *Tomentella* sp., *Hebeloma* sp., *Geopora* sp. and *Helotiales* sp., were detected on the roots of willow and birch growing in saline soils (Hrynkiewicz et al., 2015), suggesting their importance in tolerance of host-plants to salinity. Yet, the mechanism by which mycorrhizal fungi improve salt resistance remains unclear. Positive effects of *Glomus* spp. on olive tree production and growth were confirmed by different studies (Khabou et al., 2014; Mechri et al., 2014). The cultivation range of this tree crop can be limited by water scarcity as well as ubiquitous gypsum in the soil, which is responsible for osmotic stress and the ion-specific toxicity for plants (Khabou et al., 2014). A number of studies have revealed that mycorrhizal symbiosis is important for improving plant growth and nutrient uptake under saline conditions, especially the uptake of immobile soil nutrients as P, Cu, and Zn (Berruti et al., 2015). Inoculation of olive plants with *Glomus* spp. improves growth and adaptation to arid areas, although AMF colonization did not improve tolerance to Verticillium wilt, one of the most important biotic constraints affecting olive cultivation (see below), under such conditions (Kapulnik et al., 2010).

#### Endophytes and diazotrophic bacteria

Beneficial endophytes, i.e., any microbe (mainly bacteria and fungi) isolated from asymptomatic plant tissue (Hardoim et al., 2015; Brader et al., 2017) represent another taxonomically and functionally highly diverse group of microorganisms associated with tree crops. Endophytes can promote plant fitness and growth through phytohormones synthesis, nitrogen fixation, phosphate solubilization, synthesis of siderophores or reduction of ethylene levels. Some endophytes can produce active substances with biotechnological potential such as antitumor and antifungal agents (Bhore et al., 2013; Mercado-Blanco and Lugtenberg, 2014; Hardoim et al., 2015). Endophytes of tree crops can also improve the host resistance to external stresses such as contaminants, temperature extremes, water and nutrient limitations, salt, and pathogens (Mei and Flinn, 2010). Thus, it has been demonstrated that some bacterial endophytes of poplar trees can show high tolerance to trichloroethylene (TCE) and potential for degradation of these toxic compounds, e.g., *Methylobacterium populum* BJ001 (Van Aken et al., 2004), *Pseudomonas putida* W619-TCE (Weyens et al., 2010), or *Enterobacter* sp. PDN3 (Kang et al., 2012). Endophytic bacteria of willows from the phylum *Proteobacteria*, particularly the *Gammaproteobacteria*, increase considerably with cumulative contamination of soils with petroleum hydrocarbon (PHC) (Tardif et al., 2016). Finally, *Proteo*- and *Actinobacteria* from the root endosphere and from the rhizosphere of *Acer pseudoplatanus* L. show detoxifying ability in Trinitrotoluene (TNT)-contaminated soils (Thijs et al., 2014).

Diazotrophic bacteria (N_2_-fixing bacteria) are ubiquitous in the rhizosphere or inside plant tissues of both herbaceous plants and tree crops, serving as significant sources of biologically available nitrogen for them (Bagwell et al., 2001; Kandel et al., 2015). The presence of diazotrophic bacteria in plant tissues of poplar, *P. trichocarpa* (Torr. & A.Gray ex Hook.) Brayshaw, and willow, *S. sitchensis* Sanson ex Bong., including species of *Burkholderia, Rahnella, Sphingomonas*, and *Acinetobacter*, was reported by Doty et al. (2009). Experiments confirmed that inoculation of poplar with diazotrophic bacteria increases the biomass over uninoculated control plants and the growth promotion is more pronounced with multi-strain consortia than with single-strain inocula (Knoth et al., 2014). The presence of these diazotrophic microorganisms may help to explain the ability of these tree crops to grow under nitrogen limitation.

Certain trees and woody shrubs from the orders Fagales (e.g., elder, *Sambucus* sp., from *Betulaceae*, and beefwood, *Grevillea striata* R.Br., from *Casuarinaceae*), Rosales and Cucurbitales are known as “actinorhizal plants,” developing endosymbiotic relationships with filamentous, Gram-positive soil bacteria from the genus *Frankia* (*Frankiaceae*, Actinobacteria). These bacteria can fix nitrogen (N_2_) both in their free-living form and as symbionts, that is, as beneficial endophytes in root nodules developed on their host plants (Santi et al., 2013), and many actinorhizal plants form mycorrhizal associations. The host plant–*Frankia*–mycorrhiza symbiotic interaction makes these trees and shrubs capable of adapting to flooded land, arid regions, contaminated soils, extreme pH and high salinity. They can, therefore, be used for revegetation of different landscapes or for preventing desertification (Dawson, 2008; Santi et al., 2013). For example, actinorhizal plants from *Casuarinaceae* (e.g., *Casuarina equisetifolia*) have been successfully used in African coastal and desert dunes for reclamation of salt-affected soils (Diem and Dommergues, 1990).

#### Nematodes

Soil nematodes have a number of beneficial and harmful associations with tree-crops, including trophic groups which provide fundamental services in the rhizosphere. Bacteriovorous species play a key role in recycling nutrients and in the dispersal of a number of bacterial groups, including rhizobia. Some bacteriovores in Diplogasteridae may also feed on insects, whereas some Rhabditidae evolved a specialized trophism, feeding on endosymbiotic bacteria that they inoculate on insect larvae, subsequently killed by the induced sepsis. EPN and associated insect-killing bacteria are involved in the natural regulation of many insect pests. Their practical and commercial exploitation as biological control agents (BCA) has been successfully achieved in many agroecosystems, including *Citrus* and other tree crops (Lewis et al., 2015; Stock, 2015). Most important associations involve two phylogenetically distant γ-Proteobacteria, *Xenorhabdus*, and *Photorhabdus*, that evolved a close necromenic and mutualistic association with two EPN genera, *Steinernema* and *Heterorhabditis*, respectively.

**Figure 4:**
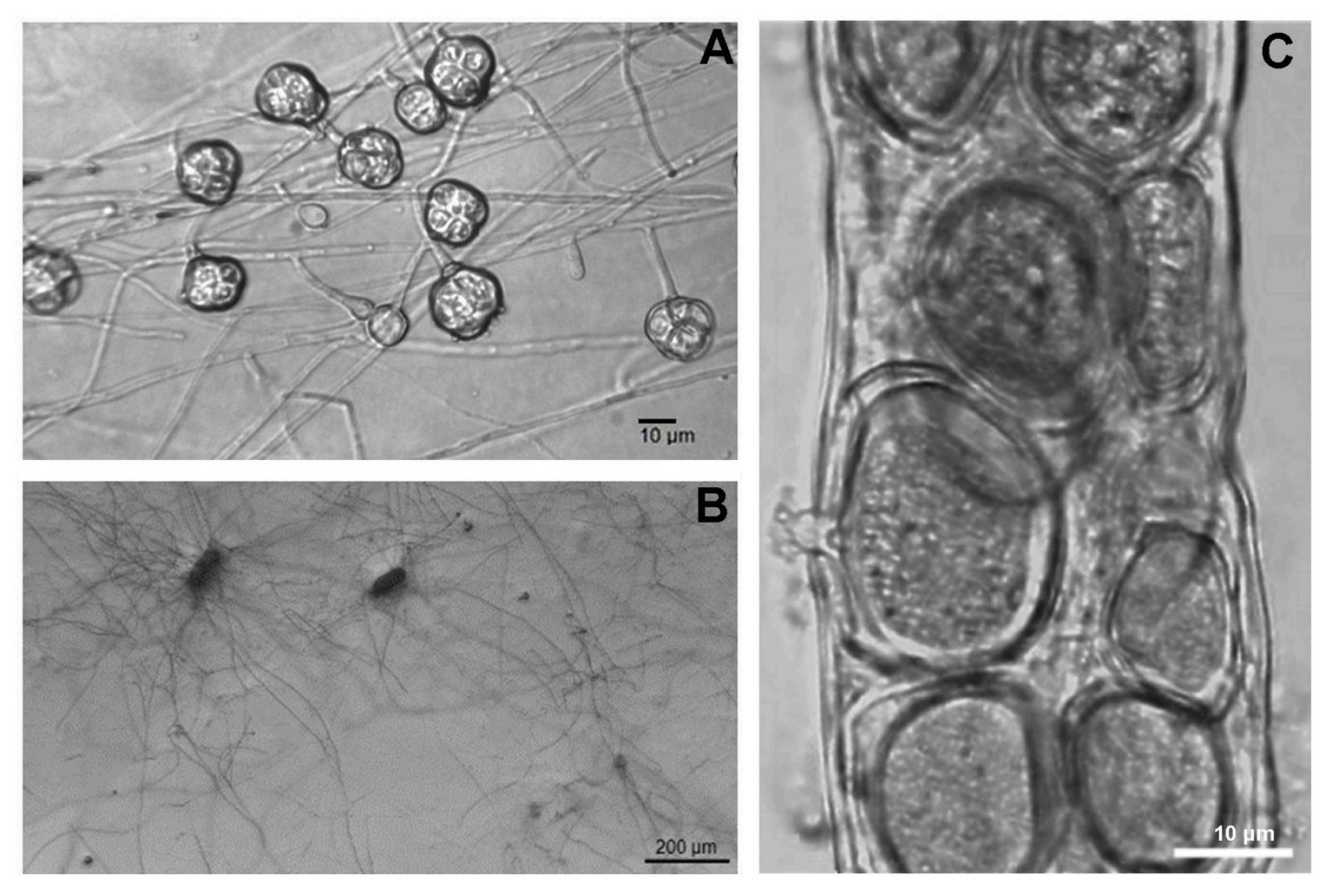
Chlamydospores of the nematode parasitic and root endophytic hyphomycete *Pochonia chlamydosporia* showing their persistent cellular structure **(A)**. Hyphae emerging from killed root-knot nematode eggs, *in vitro* **(B)**. The aquatic fungus *Catenaria anguillulae* **(C)** is one of the most common parasites of nematodes (in the picture inside *Xiphinema* sp.) killing its hosts in a few hours. However, in spite of its ubiquity and polyphagy, and due to the zoospores dependence on water for host attachment, a persistent regulation of phytoparasitic nematodes is seldom observed.

Some examples of metabolic or endosymbiotic interactions favoring trees are also available for plant-parasitic nematodes. *Pochonia chlamydosporia* (Figure 4) is a widespread hyphomycete found in soil as a facultative parasite of eggs of sedentary cyst and root-knot nematodes with a potential as a BCA. Isolates of this fungus showed different levels of adaptation to a wide range of nematode hosts, and in the ability to colonize the rhizosphere or act as root endophytes (Manzanilla-López et al., 2013). In fact, the egg parasitism seems to be correlated with *P. chlamydosporia* host preference, plant compatibility, and tolerance to abiotic factors (Vieira dos Santos et al., 2014). *Pochonia chlamydosporia* has an intimate metabolic link with roots (Rosso et al., 2014) and the potential of a *P. chlamydosporia* isolate combined with benzothiadiazole or cis-jasmonate against *M. incognita* has already been demonstrated (Vieira dos Santos et al., 2013). Studies on eggs degradation and root interactions showed changes of the fungus gene expression levels, in the transition from saprotrophic to the parasitic stage, affecting several metabolic functions. Genes activated after contact with eggs included a bZIP and a phytase-like gene. Sources of P such as phytic acid stimulated the fungal growth. Assays at varying levels of pH or glucose and ${\text{NH}}_{4}^{+}$ also showed early changes in the fungus metabolism (Rosso et al., 2011, 2014).

Data indicate that *P. chlamydosporia* plays a role in plant nutrition. Both nematode parasitism and nutrient mobilization are indicative of multiple potential benefits related to this fungus. Gene expression data on colonized barley, *Hordeum vulgare* L., revealed the production of many enzymes such as proteases, hydrolases and carbohydrate esterases (Larriba et al., 2014), suggesting a multilateral relationship with roots and nematodes. Considering the phylogenetic proximity of *P. chlamydosporia* to *Metarhizium* spp. (Larriba et al., 2014), with the ecology and metabolism of the latter species, some similarities may be inferred. In its endophytic phase, *Metarhizium* spp. provide to the plant nutrients subtracted by insects feeding on roots, when they are acting as entomopathogens, as shown using radio-labeled compounds (Behie et al., 2012). Although a similar behavior has not yet been demonstrated in *P. chlamydosporia*, it seems plausible that endophytism and parasitism may be part of a complex behavior, involving the transport of nutrients back to nematode-damaged roots. Further studies are needed to elucidate these patterns. In spite of the widespread occurrence of *P. chlamydosporia* in the rhizosphere of many perennial crops, no information exists on its role in the soil microbiota, either under controlled or field conditions. These studies would require long-lasting experiments on the changes in soil metagenome or root transcriptome, an effort not yet afforded.

### Negative effects

Although the belowground microbiota is crucial for the health of fruit, nut and SRF crops and timber trees, some members of soil microbial communities present in these agro-ecosystems have negative effects on their hosts (Table 2). On the one hand, the soil may contain inoculum sources of aboveground plant pathogens. On the other hand, the soil/rhizosphere microbiota also harbors a range of soil-borne plant pathogenic agents. Besides the prokaryotes *Rhizobium radiobacter* and *R. rhizogenes* (*Rhizobiaceae*, Rhizobiales, Proteobacteria, formerly known as *Agrobacterium tumefaciens* and *A. rhizogenes*, respectively) capable of inducing tumor formation in many economically relevant tree crops (Hwang et al., 2015), the most important negative effectors of tree health in the soil microbiota are fungus-like organisms (i.e., oomycetes) and higher fungi. A brief overview of the most relevant is presented below.

**Table 2:** Examples for the most relevant microorganisms affecting tree crops as soil-borne pathogens.

**Microbial pathogens**	**Host trees**	**Diseases**	**References**
**BACTERIA**			
*Rhizobium radiobacter* (syn. *Agrobacterium tumefaciens*)	Various fruit trees	Crown gall disease (tumor formation)	Hwang et al., 2015
**STRAMENOPILES**			
*Phytophthora* spp.	Various fruit and nut crops, forest trees	Various diseases including stem, root and/or collar rot, ink disease, dieback	see Supplementary Table 1.
*P. ultimum, P. aphanidermatum*	Tropical tree species	Damping off of seedlings	Augspurger and Wilkinson, 2007
*Pythium ultimum, P. vexans, P. irregulare, P. sylvaticum*	Apple (*Malus domestica*)	Apple replant disease	Tewoldemedhin et al., 2011; Shin et al., 2014
*P. vexans*	Rubber tree (*Hevea brasiliensis*)	Patch canker	Zeng et al., 2005
*P. undulatum*	*Abies procera*, Douglas fir (*Pseudotsuga menziesii*)	Root rot	Weber et al., 2004
Various *Pythium* spp.	Douglas fir	Damping off of seedlings	Weiland et al., 2013
**ASCOMYCETES**			
*Verticillium dahliae, V. albo-atrum*	Cork tree (*Quercus suber*), cherry (*Prunus* sp.), elder (*Sambucus* sp.), elm (*Ulmus* spp.), maple (*Acer* spp.), oak (*Quercus* spp.), olive (*Olea europaea*), peppertree (*Schinus molle*), pistachio (*Pistacia vera*), plum (*P. domestica*), smoke tree (*Cotinus* spp.), walnut (*Juglans* spp.)	Vascular wilt disease	Berlanger and Powelson, 2000
*Fusarium oxysporum* f. sp. *passiflorae*	Passion fruit (*Passiflora* spp.)	Vascular wilt disease	Ploetz, 2006
*Rosellinia necatrix*	Apple, apricot (*Prunus armeniaca*), avocado (*Persea* Americana), citrus (*Citrus* spp.), pear (*Pyrus* sp.)	White rot	Pérez-Jiménez, 2006
*Ophiostoma ulmi, Ophiostoma novo-ulmi*	Elm	Dutch elm disease	D'Arcy, 2000
*Cryphonectria parasitica*	Chestnut (*Castanea* spp.)	Chestnut blight	Anagnostakis, 2000
**BASIDIOMYCETES**			
*Armillaria mellea, A. ostoyae, A. luteobubalina*	Conifers, fruit, and nut trees	Root disease	Baumgartner et al., 2011
*Rhizoctonia* spp.	Conifers	Root damage and damping-off of seedlings	Mazzola, 1997
*R. solani*	Apple	Root rot	Mazzola, 1997
*Heterobasidion annosum*	Conifers	Root and butt rot disease	Asiegbu et al., 2005

#### Harmful oomycetes

*Phytophthora* spp. are fungus-like microorganisms belonging to the *Pythiaceae* family of Peronosporales (Oomycetes, Heterokontophyta, Chromalveolata) and can reproduce both asexually by chlamydospores, or flagellated zoospores moving in soil water, and sexually in the form of oospores (Erwin et al., 1983). Most of the *Phytophthora* species are considered soilborne pathogens, and several representatives of the genus are known to cause devastating economic losses to various tree crops worldwide (Supplementary Table 1). *Phytophthora* species also cause significant damage in nurseries and can be spread from infested nursery stocks into tree plantations and forests (Jung and Burgess, 2009). *Phytophthora* spp. are known to cause various diseases (e.g., root and collar rot, stem canker, branch and foliar dieback) in natural and planted forests (pine, larch, *Larix* spp. Philip Miller, cypress, family *Cupressaceae*, oak, *Quercus* spp., beech, alder, etc.), fruit and nut crops including avocado, *Persea americana* Mill., apple, pineapple, *Ananas comosus* (L.) Merr., peach, citrus, cocoa, *Theobroma cacao* L., almond, *Prunus dulcis* (Mill.) D.A. Webb, pomegranate, *Punica granatum* L., fig, *Ficus carica* L., pistachio, *Pistacia vera* L., and cinnamon, *Cinnamomum verum* J. Presl (Supplementary Table 1). Species like *Ph. alni, Ph. lateralis* or *Ph. quercina* are more specialized, while others (e.g.*, Ph. cinnamomi, Ph. niederhauserii, Ph. palmivora*, or *Ph. plurivora*) display a wide host range.

The genus *Pythium* from the *Pythiaceae* family, commonly occurring in forest nursery soils, also harbors important soilborne pathogens causing damping off of tree seedlings and root rot of mature trees. The life cycle of *Pythium* species is similar to that of *Phytophthora*. A study conducted on seedlings of Douglas-fir, *Pseudotsuga menziesii* (Mirb.) Franco, demonstrated that besides *Py. aphanidermatum, Py. irregulare, Py. debaryanum, Py. sylvaticum*, and *Py. ultimum*, the species *Py. mamillatum* can also cause seedling damping-off, while others, e.g., *Py. dissotocum, Py*. aff. *macrosporum, Py*. aff. *oopapillum, Py. rostratifingens*, may be responsible for seedling loss (Weiland et al., 2013). *Pythium ultimum* and *Py. aphanidermatum* were also known to infect seedlings of tropical tree species (Augspurger and Wilkinson, 2007). The species *Py. ultimum, Py. vexans, Py. irregulare* and *Py. sylvaticum* are associated with the worldwide occurring apple replant disease complex (Tewoldemedhin et al., 2011; Shin et al., 2014). *Pythium vexans* is a pathogen of rubber tree (*Hevea brasiliensis* Muell. Arg.) (Zeng et al., 2005), while *Py. undulatum* was identified as the causal agent of a devastating root rot disease of the Christmas tree *Abies procera* Rehd and Douglas fir [*Pseudotsuga menziesii* (Mirbel) Franco] in Northern Germany (Weber et al., 2004).

#### Deleterious fungi affecting tree crops

Among the higher fungi, important soilborne tree pathogens can be found both in Ascomycota and Basidiomycota. The most important ascomycetous soilborne pathogens causing wilt diseases of tree crops belong to the genera *Verticillium* and *Fusarium*. The economically most relevant member of the genus *Verticillium* (*Plectosphaerellaceae, incertae sedis*, Ascomycota) causing wilt diseases in tree crops is *V. dahliae* (Hiemstra and Harris, 1998; Berlanger and Powelson, 2000). Microsclerotia ensure the persistence of the fungus in soils for many years without susceptible hosts. In their presence, microsclerotia germinate in response to root exudates and the germinating hyphae penetrate the root, colonize the cortex and enter the xylem vessels, where the fungus is spread further by conidia (Pegg and Brady, 2002). Among many others, susceptible tree hosts of *V. dahliae* include elm, *Ulmus* spp., cork tree, *Quercus suber* L., elder, maple, *Acer* spp., oak, pepper tree, *Schinus molle* L., olive, smoke tree, *Cotinus* spp., cherry, plum, pistachio and walnut, *Juglans* spp. (Hiemstra and Harris, 1998).

Fusarium wilt is a vascular disease similar to Verticillium wilt. The disease is caused by members of the *F. oxysporum* species complex (FOSC, *Nectriaceae*, Hypocreales, Ascomycota), producing macro- and microconidia and chlamydospores allowing survival in the soil and plant debris. For instance, *F. oxysporum* f. sp. *passiflorae* causes wilt disease in passion fruit, *Passiflora edulis* Sims (Ploetz, 2006). Further important ascomycetous pathogens of trees include *Rosellinia necatrix* (*Xylariaceae*, Xylariales) causing white rot in several hosts including apples, apricots, avocados, pears and citruses (Pérez-Jiménez, 2006), *Ophiostoma ulmi* and *O. novo-ulmi* (*Ophiostomataceae*, Ophiostomatales), the causal agents of the Dutch elm disease (D'Arcy, 2000) and *Cryphonectria parasitica* (*Cryphonectriaceae*, Diaporthales) causing the blight of chestnut, *Castanea* spp. (Anagnostakis, 2000).

Concerning the basidiomycete fungi, the most relevant soil-borne tree pathogens from an economical point of view are the honey mushrooms from the genus *Armillaria* (*Physalacriaceae*, Agaricales, Basidiomycota), causing root diseases in fruit trees (e.g., *Citrus, Malus* and *Prunus* species), nut crops (e.g., *Juglans* spp.) and timber trees (e.g., *Abies, Picea, Pinus*, and *Pseudotsuga* spp.) in both hemispheres of the world under temperate, boreal and tropical climates (Baumgartner et al., 2011). The most virulent species are *A. mellea, A. ostoyae*, and *A. luteobubalina*. Mycelia of *Armillaria* species are able to survive for several years in woody residual roots even after the removal of infected trees, which serve as inoculum for the infection of the next crop. During their infection cycle, *Armillaria* species can grow in contact with the host in the form of rhizomorphs - root-like multicellular structures of clonal dispersal enabling the achievement of immense colony sizes (Sipos et al., 2017)- which employ a combination of mechanical force and extracellular enzymes to penetrate root bark (Baumgartner et al., 2011). The mycelium is then colonizing the cambium of the living roots, killing the root tissues and utilizing them for nutrition. The fungus forms white, thick mats of mycelia beneath the bark of infected roots. Further symptoms of the diseased plants include dwarfed foliage, wilting, premature defoliation and stunted shoots in the case of conifer hosts, while dwarfed fruits can be observed in the case of fruit and nut crops. After the death of the host, *Armillaria* switches from parasitic to saprophytic phase and persists in the rhizosphere as a white-rotting fungus (Baumgartner et al., 2011). *Rhizoctonia* species (*Ceratobasidiaceae*, Cantharellales, Basidiomycota) are worldwide-distributed soil fungi with the capability to produce sclerotia overwintering in the soil. Members of this genus bear significant plant pathogenic potential and a wide host range including conifers, where the fungus may cause root damage and damping-off of seedlings (Hietala and Sen, 1996). *Rhizoctonia solani* is known to cause root rot in apple orchards (Mazzola, 1997). Relevant soil-borne basidiomycetous tree pathogens also include *Heterobasidion annosum* (*Bondarzewiaceae*, Russulales) causing root and butt rot disease of conifers (Asiegbu et al., 2005).

## Harnessing beneficial components of belowground microbiota to sustain tree crops

The soil targets for protection of tree crop plantations by means of biocontrol approaches include bacterial and fungal pathogens, nematodes and insect larvae (Cazorla and Mercado-Blanco, 2016). Root and rhizosphere microbiota of healthy fruit, nut, and timber trees are rich and powerful sources of BCA (Aranda et al., 2011). Below we present an overview of representative examples of BCA used against relevant biotic constraints of tree crops. Regarding biocontrol approaches implemented against soil-borne pathogenic bacteria infecting trees, the success of the non-pathogenic *R. radiobacter* strain K84 (formerly known as *Agrobacterium radiobacter* K84) to control crown gall caused by pathogenic *R. radiobacter* strains (formerly known as *A. tumefaciens*) in different agroecosystems worldwide has been impressive. Interested readers can consult, for instance, the reviews by Moore (1988) and Kerr (2016).

### Biocontrol-based tools against deleterious oomycetes

Due to the substantial economic damage caused by fungus-like organisms, there is an emerging need for large-scale screening efforts and the development of biocontrol strategies against oomycete tree pathogens. Among prokaryotes, the most promizing taxa with potential as BCA of oomycetes are within the genus *Pseudomonas* (Gammaproteobacteria, Pseudomonadales, *Pseudomonadaceae*) (Mercado-Blanco, 2015) and the order Bacillales (Firmicutes) (Borriss, 2015). Examples of bacteria-based biocontrol of woody crop diseases caused by *Phytophthora* spp. include field studies performed in citrus orchards against *Ph. parasitica* using *P. putida* 06909, a biocontrol strain capable of actively colonizing the hyphae of *Phytophthora* spp. (Steddom et al., 2002). Acebo and colleagues isolated 127 rhizobacteria from the rhizosphere of cocoa, identifying three strains of *P. chlororaphis* with both *in vitro* and direct antagonistic potential against the black pod rot pathogen *Ph. palmivora*. The biosurfactant viscosin was found to be crucial for the motility and biofilm formation of *P. chlororaphis*. Even though the involvement of viscosin in antagonism against *Phytophthora* was not demonstrated, its possible role in the bioprotection of *T. cacao* was suggested (Acebo-Guerrero et al., 2015). The ability of *Bacillus amyloliquefaciens* (Firmicutes, Bacillales, *Bacillaceae*) strain HK34 to induce systemic resistance in ginseng to *Ph. cactorum* suggests that this species may have potential also in the management of other tree diseases caused by the same pathogen (Lee et al., 2015).

Besides bacteria, the ascomycete *Trichoderma* (Hypocreales, *Hypocreaceae*) is also a powerful source of potential BCA against oomycete tree pathogens. Thus, the mycoparasitic activity of *T. virens* was shown to be involved in the control of *Pythium. ultimum* (Djonović et al., 2006), while the antagonistic potential of strains *T. virens* T7, *T. harzianum* T40, *T. asperellum* T54 and *T. spirale* T4 was demonstrated against *Ph. palmivora* (Mpika et al., 2009). *Trichoderma saturnisporum* was recently found to improve plant quality and showed biocontrol activity against *Phytophthora* spp., including *Ph. parasitica* (Diánez Martínez et al., 2016).

### Biological control of soil-borne phytopathogenic fungi causing vascular diseases

Soil-borne fungi causing vascular diseases are also important threats to plants, including woody hosts. Pathogenic representatives of *Verticillium* spp. pose a serious risk in many agro-ecosystems worldwide (Pegg and Brady, 2002; Inderbitzin et al., 2011). Verticillium wilts are among the most threatening biotic constraints for tree crops in many areas (Hiemstra and Harris, 1998). Biological control exerted by soil-borne beneficial microorganisms can be useful to confront the disease, particularly when applied as a preventive measure (Mercado-Blanco et al., 2004). One of the best examples in which effective BCA have been identified, characterized and successfully used is the case of Verticillium wilt of olive (VWO) caused by *V. dahliae* Kleb (López-Escudero and Mercado-Blanco, 2011). Strains of *Pseudomonas* spp. have been isolated from the olive rhizosphere (and elsewhere), and proved to suppress VWO in young, nursery-produced plants (Mercado-Blanco et al., 2004; Sanei and Razavi, 2011; Triki et al., 2012; Gómez-Lama Cabanás et al., 2018). One of the best known BCA against VWO is *P. fluorescens* PICF7 (Prieto et al., 2009; Martínez-García et al., 2015). This strain is a natural inhabitant of the olive rhizosphere and endophytically colonizes olive root tissues (Prieto and Mercado-Blanco, 2008; Prieto et al., 2011). While our knowledge about the traits of strain PICF7 involved in both endophytism and biocontrol is scarce (Maldonado-González et al., 2015), results have shown that olive root colonization by this bacterium triggers broad transcriptomic changes, both at local (roots) and systemic (aboveground tissues) level (Schilirò et al., 2012; Gómez-Lama Cabanás et al., 2017). Many of these changes are related to defense responses to different (a)biotic stresses and may shed light on why this endophyte is recognized by the host as a non-hostile colonizer and provide clues on the underlying mechanisms of its biocontrol activity. However, while aboveground defense responses are induced upon strain PICF7 root colonization, they are not effective to control another relevant olive pathogen, *Pseudomonas savastanoi* pv. *savastanoi* causing olive knot disease (Maldonado-González et al., 2013). Furthermore, where and when strain PICF7 is applied in the olive root system seems to be crucial for the effective suppression of VWO (Gómez-Lama Cabanás et al., 2017). Other soil-borne microorganisms have been studied and used as effective antagonists and/or BCA against *V. dahliae*, such as the bacteria *Serratia plymuthica* HRO-C48 (Müller et al., 2007) and *Paenibacillus alvei* K165 (Markakis et al., 2016), or the fungi *T. harzianum* CECT 2413 (Ruano-Rosa et al., 2016) and *T. asperellum* T25 and Bt3 (Carrero-Carrón et al., 2016). The report by Markakis et al. (2016) demonstrated for the first time an effective biocontrol of VWO under field conditions, a scenario not frequently explored in biocontrol research, particularly with trees (Cazorla and Mercado-Blanco, 2016). A recent review highlights all desirable traits that a BCA should have to confront pathogenic *Verticillium* spp., including those ones affecting tree crops. Similar requisites can likely be taken into account, when considering other soil-borne fungal phytopathogens (Deketelaere et al., 2017).

Additional prominent examples of biological control of tree pathogenic ascomycetes are the application of *V. albo-atrum* for the control of Dutch elm disease caused by *O. ulmi* and *O. novo-ulmi* (Scheffer et al., 2008; Postma and Goossen-van de Geijn, 2016), the exploitation of the hypovirulence phenomenon in the case of a dsRNA mycovirus-harboring strain of *C. parasitica* against chestnut blight (Milgroom and Cortesi, 2004) or the possibility of using fungi (*Trichoderma* species) or bacteria (*P. fluorescens, Bacillus subtilis*) for the control of avocado white root rot caused by *R. necatrix* (Sztejnberg et al., 1987; Cazorla et al., 2006, 2007; Ruano-Rosa and López Herrera, 2009).

### Biological control of other phytopathogenic fungi

Amongst the soilborne basidiomycete pathogens of fruit and nut crops and timber trees, the main targets of biocontrol efforts are members of the genus *Armillaria*. BCA of *Armillaria* act through the limitation of the pathogen to—or elimination from—the already occupied substrate, and prevention of rhizomorph and mycelium development (Fox, 2003). Potential *Armillaria* antagonists include *Trichoderma* species: scanning electron microscopy studies revealed that some *Trichoderma* strains are able to attack and penetrate the outer tissue of the rhizomorphs, killing *Armillaria* hyphae after coiling and direct penetration (Dumas and Boyonoski, 1992; Pellegrini et al., 2012). Other fungi antagonistic to *Armillaria* include *Rhizoctonia lamellifera* that prevents the pathogen from colonizing tea roots, *Scytalidium lignicola* and its toxin scytalidin inhibiting *Armillaria* growth *in vitro, Phlebiopsis gigantea* and *Pleurotus ostreatus* capable of excluding *Armillaria* from its substrates, *Coriolus versicolor, Stereum hirsutum*, and *Xylaria hypoxylon* reducing the stump colonization by *Armillaria*, and cord-forming saprotrophs acting as competitive antagonists (Fox, 2003). The method based on isotope ratio mass spectrometry developed to study trophic interactions between *A. mellea* and fungal/bacterial antagonists is a promizing tool for the screening of further potential BCA (Pellegrini et al., 2012). Further examples for the biological control of tree pathogenic basidiomycetes are the application of forest soil-derived *Streptomyces* spp. or *P. gigantea* (Basidiomycota, Polyporales, *Phanerochaetaceae*) to control *H. annosum* causing root and bud rot of conifers (Lehr et al., 2008; Sun et al., 2009).

### Biological control strategies against nematode and insect pests

Some specific and effective nematode antagonists such as *Pasteuria* spp. have been reported on tree crops, and their regulatory role described as well (Ciancio, 1995; Ciancio et al., 2016). As concerns the role of bacteria in nematode and insect management (see below), it is worth mentioning that our knowledge about several lineages is still very limited (Roesch et al., 2007).

In most cases, nematodes play different roles in soil food webs, acting as preys, predators, saprotrophs, or feeding on bacteria, fungi, roots or other invertebrates (Figure 2). Their association with tree roots and endoparasites, such as *Pasteuria* spp., can be monitored through the collection of time series data on host density and prevalence. *Pasteuria* spp. have a very narrow host specificity, due to an obligate parasitic behavior. Their persistence in soil is due to the presence of durable endospores, which are also the infective propagules. Through this strategy these bacteria reduce their competition with other soil bacteria, confining their vegetative growth in the small microhabitat provided by the nematode body. This food web can persist for 20 years, as experimentally shown on a citrus grove in Southern Italy (Ciancio et al., 2016). In a different study on *Xiphinema diversicaudatum*-peach and *Pasteuria* sp. carried out in Piedmont, the food web persisted for at least 15 years. The nematode is a virus vector, and its population was also targeted by a predatory nematode (*Discolaimus* sp.), which in turn hosted a distinct *Pasteuria* sp. After trees have been removed from the parcel of study, the nematodes and *Pasteuria* associations were found 20 years later in other adjacent fields, suggesting a local endemism due to soil movement by farmers or water flows, and to the presence of natural reservoirs.

Until the late 1980's, many nematode pests were mostly managed by pesticides or soil fumigants. However, the use of nematicides raised several concerns for their potential harm to farmers, consumers, and damage to the environment (wildlife, water or soil pollution). Attention has thus been given to the effects of biological components of the rhizosphere on nematodes. Bacterial and fungal components of tree rhizosphere microbiota can also be exploited as BCA of phytoparasitic and soil-dwelling nematodes and insect larvae damaging forests and tree plantations. Predation and parasitism arose several times during the evolution of early eukaryotes and may be found among aquatic fungi, ascomycetes, and basidiomycetes. Aquatic fungi such as *Catenaria anguillulae* or *Myzocitium* spp. penetrate the nematode cuticle through motile zoospores that adhere to the host. After an encystation stage, colonization of the host body occurs through germinating thalli. While these species have specific parasitic habits and can regulate nematodes in a humid and wet soil environment, their regulatory potential appears, however, limited depending on high soil water content (Singh et al., 2007).

Many hyphomycetes like *Arthrobotrys* or *Drechslerella* spp. (Ascomycota, *Orbiliaceae*) produce hyphal traps or nets that actively capture and/or attract passing nematodes. This character arose through adaptive evolution in two distinct lineages, one trapping through constricting rings and the other by adhesive nets (Yang et al., 2007). Other parasitic strategies developed by hyphomycetes include the direct, passive adhesion of infective conidia to the nematode cuticle, with germinating hyphae penetrating the host to develop a lethal infection. These strategies are found in species such as *Hirsutella rhossiliensis* (anamorph of *Cordiceps* sp.), *Meria coniospora* or *Nematoctonus* spp., the latter a teleomorph of *Hohenbuehelia* (Basidiomycota, Agaricales, *Pleurotaceae*). *Nematoctonus* also shows the production of toxins by the germinating conidia, which reduce the host movement, thus lowering the probabilities of an early loss of the infective propagule (Giuma and Cooke, 1971). *Paecilomyces* (*Purpureocillium*) *lilacinus* may degrade nematode eggs and regulate their density, due to the activity of several chitinolytic and proteolytic enzymes. The latter provides the fungus a strong keratinolytic activity, a trait supporting its pathogenicity to superior animals, including man.

*Pochonia chlamydosporia* is also a root endophyte that may elicit several defensive pathways after colonization, without induction of any visible root damage (Maciá-Vicente et al., 2009; Ciancio et al., 2013; Rosso et al., 2014; Larriba et al., 2015). This behavior is indicative of a long-term evolutionary adaptation to the rhizosphere environment, exploiting strategies involving multitrophic relationships with the plants and other rhizosphere organisms.

Finally, pine wilt disease is caused by the pinewood nematode *Bursaphelenchus xylophilus*, leading to the death of susceptible pine trees. In order to control this disease, a few studies have been performed using chemical or biological compounds (Proença et al., 2017b). Several strains were reported to produce extracellular compounds with nematicidal activity, among which *Serratia marcescens* A88copa13 that produces an extracellular serine protease as the major key factor toward the nematode (Paiva et al., 2013).

Although most of the insect damage to fruit and nut crops and forest trees can be attributed to their herbivoural defoliating activity, a few of them are also important as soil-borne pests because their larvae feeding on the roots. An example of EPN impact and the regulatory role played in soil food webs is the biocontrol and management of *Diaprepes* sp. and other root-weevils infesting citrus and other perennial crops in Florida (Campos-Herrera et al., 2013, 2015). Other relevant examples are the larvae of May bugs (also known as white grubs), especially those of the forest cockchafer (*Melolontha hippocastani*), a species widely distributed in Eurasia. Besides EPN like Steinernematidae and *Heterorhabditis* spp. (Woreta, 2015), larvae of the forest cockchafer are subjected to infections by entomopathogenic fungi (e.g., *Beauveria brongniartii*) and bacteria (like *Bacillus popilliae* var. *melolonthae* or *B. thuringiensis*).

In the case of *B. brongniartii*, cereal grains infected with mycelia is the most frequent formulation used for the control of *M. hippocastani*. However, as summarized by Woreta (2015), the field performance of this biocontrol strategy revealed ambiguous results during several attempts since the 1880s in France, Poland, Italy, Switzerland, and Germany. This situation can be explained by difficulties of introducing and blending infected grains with the soil, especially around young trees where the abundance of cockchafer grubs is expected. Although it was shown that, under field conditions, grub population can be decreased to a harmless level by the application of an adequate *B. brongniartii* formulation thoroughly mixed with soil and applied at sufficient air temperature and humidity, *B. brongniartii* has not been authorized in the EU for use in commercial plant protection products (Woreta, 2015).

Among bacteria, *B. popilliae* var. *melolonthae*, the causal agent of the milky disease, has also been studied as a potential BCA of cockchafer grubs (Franken et al., 1996). The disease incidence increased when the grubs were infected simultaneously with *B. popilliae* and *B. brongniartii*, which is possibly due to synergistic effects between the two pathogens, suggesting the possibility of integrated biological control. Highly pathogenic *B. thuringiensis* subsp. *tenebrionis* and *B. weihenstephanensis* strains, isolated from larvae of the common cockchafer *M. melolontha* (Kati et al., 2007; Sezen et al., 2007), or *Serratia* species, causing feeding discontinuation of *M. hippocastani* larvae (Jackson and Zimmermann, 1996), may be valuable as BCA of cockchafer white grubs damaging tree roots.

### Inconsistencies and risk assessment in biological control of tree crops

Inconsistent field performance is one of the major challenges in the application of beneficial microorganisms as BCA and/or plant growth promoters (Weller et al., 1995). It is even more complex in the case of trees because of their own idiosincracy (Cazorla and Mercado-Blanco, 2016). Inconsistency can be the result of various abiotic and biotic factors (Meyer and Roberts, 2002). Physicochemical properties of the rhizosphere (temperature, pH, water availability, chemical composition) are parameters varying both in space and time, which have substantial influence on the performance of plant growth promoting and biocontrol microorganisms: an individual agent can have different activities in different soil environments. One of the possible approaches to counteract inconsistencies under different environmental conditions is the development of strategies based on more than just a single beneficial organism. The combined application of wide-spectrum BCA with efficient plant growth promoting microorganisms has the potential to reach the increased consistency of performance over a wider range of soil conditions. A recent example was presented by Imperiali et al. (2017), who applied *Pseudomonas* bacteria, AM fungi and EPN to improve wheat performance. Moreover, the application of entire, well-characterized, complex microbiota may further improve the efficiency of soil-borne pathogen management and other biotic constraints (Gopal et al., 2013; Berg et al., 2014; Kowalski et al., 2015). Other examples are the effect of chemically and microbiologically characterized vegetable compost in oak seedlings on decline caused by *Ph. cinnamomi* (Moreira et al., 2010), and the efficiency of organic amendments (yard waste and almond shells) to avocado crops in suppression of the white root rot fungus, *R. necatrix* (Bonilla et al., 2015). Based on their results these authors suggested that organic amendments can be useful cultural practices to reduce the impact of the pathogens.

Although sophisticated and ecologically “intelligent”, many fungi acting as predators or parasites show a reduced biocontrol efficacy for pests such as root-knot (*Meloidogyne* spp.), cyst (*Heterodera* spp., *Globodera* spp.) or other nematode species, once applied to soil as bioformulations (Jaffee, 1992; Kluepfel et al., 2002; Castillo et al., 2010). The reasons for such low performance may depend on several factors, including the inhibition by the resident soil microflora, the evolution of low virulence traits allowing the maintenance of the host population, or the capacity of most fungi to grow on a wide range of substrates, using nematodes as additional food sources. Other factors are related to density-dependent relationships established with their hosts, as shown for *H. rhossiliensis* on *M. xenoplax* on peach or for other fungi parasitic on nematode eggs on kiwi (Jaffee et al., 1989; Roccuzzo et al., 1993). A further factor concerns the evolution of more complex adaptative behaviors, as in the case of the egg parasite *P. chlamydosporia* (Figure 4). This parasite produces specific enzymes allowing the lysis of the egg cuticle and vitelline layers, a step followed by the egg colonization through an appressorium and growing hyphae. This fungus has been reported as a highly-effective BCA, displaying specificity for the nematode species from which the isolates were obtained (Manzanilla-López et al., 2013).

Lastly, when planning the application of a biocontrol strategy, a thoroughly performed risk assessment is necessary. The EU policy support action REBECA (Regulation of Biological Control Agents) aims to review the possible risks of biocontrol agents (http://www.rebeca-net.de/?p=999). BCA may have negative effects on beneficial, non-target organisms (e.g. mycorrhizal fungi) or other crops. For example, although many *Trichoderma* species are considered as potential BCA for the protection of both herbaceous and woody plants, certain members of the genus, e.g., *T. aggressivum*,*T. pleurotum* and *T. pleuroti*, represent a risk to commercial mushroom production where they can cause green mold disease (Hatvani et al., 2008; Kredics et al., 2010) or to human health, with *T. longibrachiatum* as a potential opportunistic human pathogen (Hatvani et al., 2013). The application of these *Trichoderma* species for biocontrol purposes should, therefore, be carefully monitored.

### Coping with abiotic stresses and phytoremediation

Tree crops used in SRF aiming to biomass production (e.g., *Salix* spp. and *Populus* spp. and their hybrids) have been successfully used as sustainable solutions to recover contaminated soil (Licht and Isebrands, 2005; Zalesny et al., 2016). Phyto-assisted bioremediation, or phytoremediation, is an *in situ* treatment of contaminated soils, which relies on complex interactions established between roots and soil microorganisms in the rizhosphere (Wenzel, 2009). In this microhabitat, bacterial communities can respond promptly to pollutant occurrence, promoting organic contaminant degradation and/or inorganic phyto-containment (Simpson et al., 2009). Bioaugmentation of soils with selected microorganisms can significantly increase efficiency of phytoremediation (Złoch et al., 2017). The synergistic action between the tree root system and the natural belowground microbiota makes it possible to remove, convert, or contain toxic substances in soils.

Beyond the contaminant removal, an overall soil quality improvement is observable in terms of soil carbon sequestration, increased nutrient content, recycling and biomass production for energy purposes. Poplar is one of the most used tree crops for stimulating (e.g., through root exudates production, oxygen transport) bacterial degradation of persistent organic contaminants (e.g., polychlorinated biphenyls - PCB) and phyto-containment of inorganic ones (heavy metals) in the rhizosphere (Gamalero et al., 2012; Ancona et al., 2017). However, other tree species have been successfully applied for this purpose such as willow (*Salix* spp.), eucalyptus, black locust (*Robinia pseudoacacia* Simpson et al., 2009) and *Corylus* spp. for metal and metalloid phyto-containment (Radojevic et al., 2017). Although bacteria and archaea are the only groups within the plant microbiota able to transform and mineralize organic contaminants, their huge metabolic potential remains to be explored.

## Concluding remarks: toward microbiota-assisted management strategies

**Figure 5:**
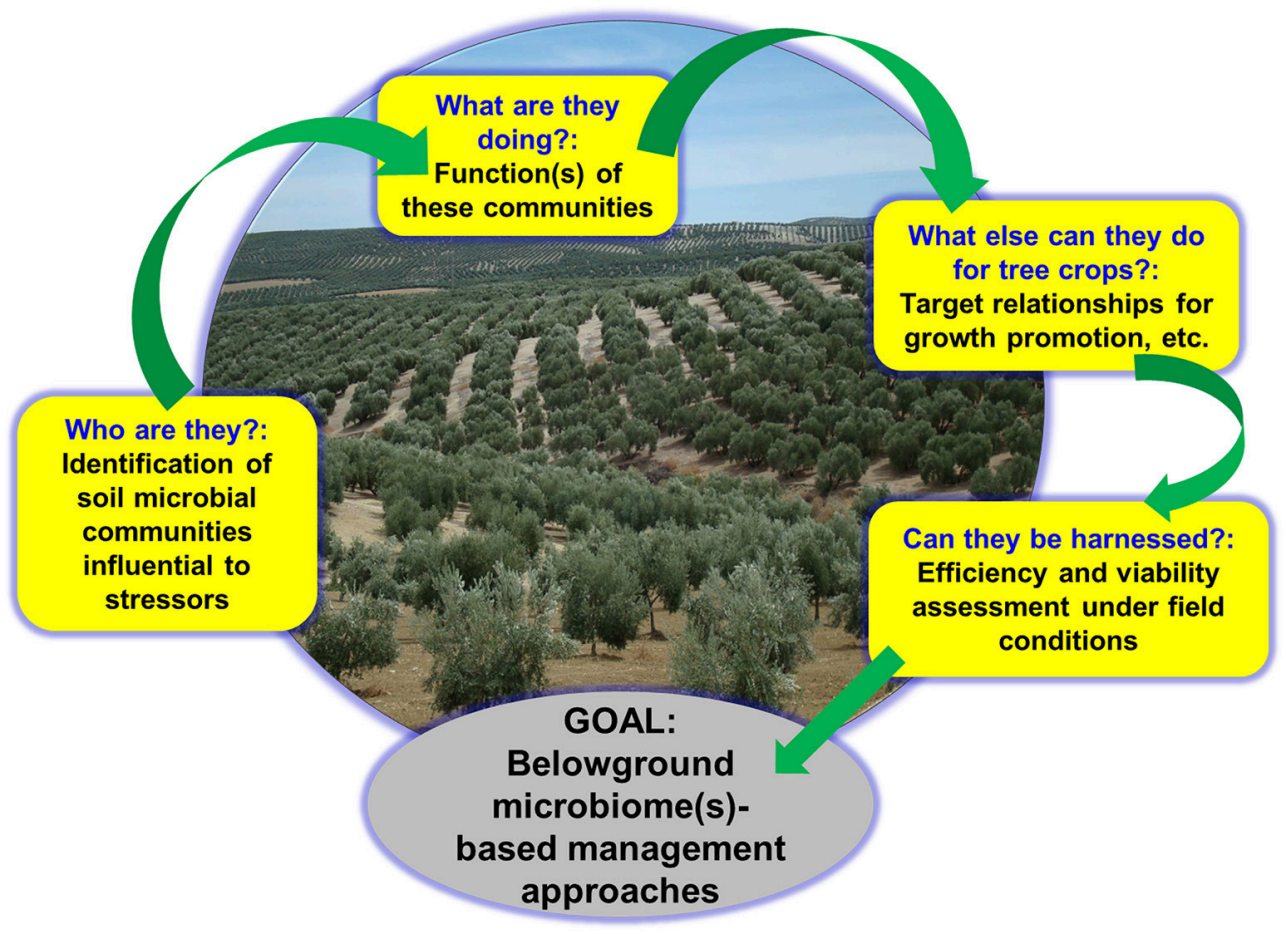
A strategy to manage biotic constraints affecting tree crops (i.e., pathogens, pests, invasive species) based on the identification, characterisation and harnessing of soil/root microbiota [based on a conceptual framework by Kowalski et al. (2015)].

Belowground microbial communities associated with tree crops are key factors for their growth, development, and health, particularly under non-favorable soil conditions. They decisively contribute to enhanced productivity, improve accessibility to low-abundant nutrients, cope with a range of (a)biotic stressors that affect their associated hosts, and also play an important role in phyto-assisted biodegradation of toxic compounds present in soils. Until now, how belowground microbiota contribute to the fitness of tree crop agro-ecosystems, remains largely unknown and only now it is starting to be unraveled in detail. The four fundamental questions to better understand these associations are: *who are there? what are they doing? who is active out there?* and *how do activities of these microorganisms relate to ecosystem functions?* (Amann, 2000; Leveau, 2007). The answers to these questions, based on an in-depth knowledge of the structure and functioning of belowground communities, will constitute the pillars to develop holistic management strategies aiming to cope with the range of (a)biotic constraints affecting tree crops (Figure 5). The relationship between soil-borne microbes and tree crops is delicate and complex and can have either positive or negative effects on the host. It can be assumed that benefits derived from the interaction of tree crops with beneficial belowground (micro)organisms are expected to yield similar outcomes in aboveground ecosystems than those observed, and more frequently investigated, in herbaceous, short-living species. Moreover, the associations established with trees are expected to be more stable, enduring along time, although variations in composition, structure, and functioning do occur, likely in a cyclic manner. These are subjected to a broad range of genetic, (a)biotic and environmental cues and factors. In this sense, integrated “omic” analyses, combining metagenomics, metatranscriptomics, metaproteomics, and metabolomics, are now providing a more accurate view of the activities and the physiological potential of belowground plant-associated microbiota (Zhang et al., 2010; Knief, 2014).

Studies on tree crop production and diseases have thus far historically relied on single microbe-based formulations or focused on single species (the pathogen), while little attention has been paid to the use of consortia of beneficial microorganisms or to investigate many other microorganisms most likely present in the infection sites. One way to assist tree crop production might be to integrate beneficial plant microbiota or use *ad hoc* tailored microbiota to target specific deleterious agents (Gopal et al., 2013; Kowalski et al., 2015; Pinto and Gomes, 2016; Berg et al., 2017; Figure 5). Due to the complexity of tree crop ecosystems—dominated by vegetal species displaying peculiarities such as large biomass, complicated anatomy, large root systems, longevity, and the large spatial domains and timescales over which tree crops are grown –management options such as soil amendments, intercropping and soil processing can be applied by farmers. Once again, the currently-available multi-omic tools, combined with other methodological approaches, will provide a much better knowledge on the complex network of trophic interactions taking place at the soil/root level (Massart et al., 2015). A more-in-depth analysis of these interactions could be of crucial importance in designing new and effective microbial consortia for optimizing plant production and developing new strategies for disease control. In conclusion, a more holistic approach to tree crop agriculture is needed. Understanding the microbial diversity, distribution, activity, and function, and linking the microbial community structure with both environmental factors and ecosystem functioning, are major challenges for the soil/plant microbiology science in this century.

## Author contributions

All authors listed have made a substantial, direct and intellectual contribution to the work, wrote the review and gave approval to the final version. JM-B designed the study. AB critically reviewed the manuscript and supervised the manuscript drafting.

### Conflict of interest statement

The authors declare that the research was conducted in the absence of any commercial or financial relationships that could be construed as a potential conflict of interest.
