# Supplementary material for: Belowground Microbiota and the Health of Tree Crops
Source: Front Microbiol. 2018 Jun 5;9:1006. doi: 10.3389/fmicb.2018.01006 (PMC5996133; doi:10.3389/fmicb.2018.01006)
Supplement: Supplementary file 1 [file Table_1.pdf]

Supplementary Material

Belowground Microbiota and the Health of Tree Crops

Jesús Mercado-Blanco[1], Isabel Abrantes[2], Anna Barra Caracciolo[3], Annamaria Bevivino[4*], Aurelio Ciancio[5], Paola Grenni[3], Katarzyna Hrynkiewicz[6], László Kredics[7], Diogo N. Proença[8]

*** Correspondence:** Dr. Prof. Annamaria Bevivino: annamaria.bevivino@enea.it

1 Supplementary Table

Supplementary Table 1. *Phytophthora* species affecting tree crops as soil-borne pathogens

Supplementary Table 1. *Phytophthora* species affecting tree crops as soil-borne pathogens

***Phytophthora* species**	**Host trees**	**Diseases**	**Geographic region**	**References**
Ph. alni	Alder (*Alnus* spp.)	Decline	Europe	(Aguayo et al. 2014)
Ph. austrocedri	*Austrocedrus chilensis*	Dieback	Argentina	(Mulholland et al. 2015)
	Juniper (*Juniperus communis*)	Mortality	Northern England	(Mulholland et al. 2015)
Ph. cactorum	Apple (*Malus* spp.)	Apple replant disease	South Africa	(Tewoldemedhin et al. 2011)
Ph. cambivora	European chestnut (*Castanea sativa*)	Ink disease	Europe	(Robin et al. 2006)
Ph. cinnamomi	Jarrah (*Eucalyptus marginata*)	Dieback	Australia	(Shearer and Tippett, 1989)
	Pine (*Pinus* spp.)	Little leaf disease	Scotland	(Tainter, 1997)
		Growth reduction		(Chavarriaga et al. 2007)
	Avocado (*Persea americana*)	Root rot, branch dieback	World wide	(Reeksting et al. 2016)
	Avocado, pineapple, peach, chestnut, oak, eucalyptus	Root rot, stem canker	World wide	(Hardham, 2005)
Ph. cryptogea	European chestnut	Ink disease	Europe	(Perlerou et al. 2010)
Ph. katsurae	Japanese chestnut (*Castanea crenata*)<br>Coconut (*Cocos nucifera*)	Ink disease<br>Heart rot	Japan, South Korea<br>Hawaii, Côte d'Ivoire	(Oh and Parke, 2012)
Ph. lateralis	Lawson cypress (*Chamaecyparis lawsoniana*)	Dieback	Southwest Oregon, Northern	(Hansen 1999)<br>(Mulholland et al.

			California, UK	2015)
Ph. megakarya	Cocoa (*Theobroma cacao*)	Pod rot	West Africa	(Opoku et al. 2000)
Ph. niederhauserii	Arborvitae (*Thuja occidentalis*)<br>Leyland cypress (*Cupressocyparis leylandii*)<br>Juniper (*Juniperus* sp.)<br>Almond (*Prunus dulcis*)<br>Pomegranate (*Punica granatum*)<br>Nordmann fir (*Abies nordmanniana*)<br>Fig (*Ficus carica*)<br>Indonesian cinnamon (*Cinnamomum burmannii*)<br>Avocado<br>Pistachio (*Pistacea vera*)	Root, crown and stem symptoms	USA<br>USA<br>USA<br>Spain<br>Spain<br>Hungary<br>Taiwan<br>Indonesia<br><br>USA, Israel<br>Iran	(Abad et al. 2014)
Ph. palmivora	Cocoa	Pod rot	Pantropical	(Drenth and Guest, 2013)
	Citrus (*Citrus* sp.)<br>Durian (*Durio zibethines*)<br>Jackfruit (*Artrocarpus heterophyllus*)<br>Rubber (*Hevea brasiliensis*)<br>Coconut	Various diseases	Pantropical	
Ph. parasitica	Citrus	Brown rot, foot rot, gummosis, root rot	California	(Rosa et al. 2007)
Ph. parsiana	Pistachio<br>Fig<br>Almond	Decay	Iran, USA<br>Iran<br>Greece	(Mostowfizadeh-Ghalamfarsa et al. 2008)
Ph. plurivora	Beech (*Fagus sylvatica*)<br>Oak (*Quercus robur*)<br>Norway maple (*Acer platanoides*)<br>Small-leaved linden (*Tilia cordata*)<br>Norway spruce (*Picea abies*)	Root rot	Europe, North America	(Jung and Burgess, 2009)

Ph. pseudosyringae	Oak (*Quercus* spp.)<br>Beech<br>Alder (*Alnus glutinosa*)	Collar rot	Europe	(Jung et al. 2003)
Ph. psychrophila<br>*Ph. uliginosa*<br>*Ph. europaea*	European oak (*Quercus* spp.)	Oak decline	Europe	(Jung et al. 2002)
Ph. quercina	Oak	Root rot	Europe	(Jung et al. 1999)
Ph. ramorum	Oak	Sudden oak death	Europe, North America	(Goheen et al. 2002)
	Larch (*Larix kaempferi*)	Needle necrosis	UK	(Mulholland et al. 2015)
Ph. syringae	Apple<br>Pear (*Pyrus communis*)	Stem and root rot	USA	(Laywisadkul et al. 2010)

References:

Abad, Z. G., Abad, J. A., Cacciola, S. O., Pane, A., Faedda, R., Moralejo, E., et al. (2014). *Phytophthora niederhauserii* sp. nov., a polyphagous species associated with ornamentals, fruit trees and native plants in 13 countries. Mycologia 106, 431–447. doi: 10.3852/12-119

Aguayo, J., Elegbede, F., Husson, C., Saintonge, F.-X., and Marçais, B. (2014). Modeling climate impact on an emerging disease, the *Phytophthora alni* induced alder decline. Glob. Chang. Biol. 20, 3209–3221. doi: 10.1111/gcb.12601

Chavarriaga, D., Bodles, W. J. A., Leifert, C., Belbahri, L., and Woodward, S. (2007). *Phytophthora cinnamomi* and other fine root pathogens in north temperate pine forests. FEMS Microbiol. Lett. 276, 67–74. doi: 10.1111/j.1574-6968.2007.00914.x

Drenth, A., and Guest, D. (2013). *Phytophthora*: a Global Perspective. (Wallingford: CABI). doi: 10.1079/9781780640938.0187.

Goheen, E. M., Hansen, E. M., Kanaskie, A., McWilliams, M. G., Osterbauer, N., and Sutton, W. (2002). Sudden oak death caused by *Phytophthora ramorum* in Oregon. Plant Dis. 86, 441–441. doi: 10.1094/PDIS.2002.86.4.441C

Hansen, E. (1999). "*Phytophthora* in the Americas," in *Phytophthora Diseases of Forest Trees*, Proceedings from the First International Meeting on Phytophthoras in Forest and Wildland Ecosystems, ed E. M. Hansen and W. Sutton (Oregon), 23–27.

Hardham, A. R. (2005). *Phytophthora cinnamomi*. Mol. Plant Pathol. 6, 589–604. doi:

10.1111/j.1364-3703.2005.00308.x

Jung, T., Cooke, D. E. L., Blaschke, H., Duncan, J. M., and Oßwald, W. (1999). *Phytophthora quercina* sp. nov., causing root rot of European oaks. Mycol. Res. 103, 785–798. doi: 10.1017/S0953756298007734

Jung, T., Hansen, E. M., Winton, L., Oswald, W., and Delatour, C. (2002). Three new species of *Phytophthora* from European oak forests. Mycol. Res. 106, 397–411. doi: 10.1017/S0953756202005622

Jung, T., Nechwatal, J., Cooke, D. E. L., Hartmann, G., Blaschke, M., Oßwald,W. F., et al. (2003). *Phytophthora pseudosyringae* sp. nov., a new species causing root and collar rot of deciduous tree species in Europe. Mycol. Res. 107, 772–789. doi: 10.1017/S0953756203008074

Jung, T., and Burgess, T. I. (2009). Re-evaluation of *Phytophthora citricola* isolates from multiple woody hosts in Europe and North America reveals a new species, *Phytophthora plurivora* sp. nov. Persoonia. Mol. Phylogeny Evol. Fungi 22, 95–110. doi: 10.3767/003158509X442612

Laywisadkul, S., Fuchigami, L. H., Scagel, C. F., and Linderman, R. G. (2010). Tree growth stage and environment after pathogen inoculation alters susceptibility of pear trees to Phytophthora canker. Horticulture 3, 11–20. doi: 10.2174/1874840601003010011

Mostowfizadeh-Ghalamfarsa, R., Cooke, D. E. L., and Banihashemi, Z. (2008). *Phytophthora parsiana* sp. nov., a new high-temperature tolerant species. Mycol. Res. 112, 783–794. doi: 10.1016/j.mycres.2008.01.011

Mulholland, V., Elliot, M., and Green, S. (2015). Diagnostics of tree diseases caused by *Phytophthora austrocedri* species. Methods Mol. Biol. 1302, 59–73. doi: 10.1007/978-1-4939-2620-6_5

Oh, E., and Parke, J. L. (2012). *Phythophthora katsurae*. Forest Phytophthoras 2. doi: 10.5399/osu/fp.2.1.3046

Opoku, I. Y., Appiah, A. A., Akrofi, Y., and Owusu, G. K. (2000). *Phytophthora megakarya*: a potential threat to the cocoa industry in Ghana. Ghana J. Agric. Sci. 33, 237–248. Available online at: www.ajol.info/index.php/gjas/article/view/1876

Perlerou, C., Tziros, G., Vettraino, A. M., and Diamandis, S. (2010). *Phytophthora cryptogea* causing ink disease of *Castanea sativa* newly reported in Greece. Plant Pathol. 59, 799. doi: 10.1111/j.1365-3059.2010.02268.x

Reeksting, B. J., Olivier, N. A., and van den Berg, N. (2016). Transcriptome responses of an ungrafted *Phytophthora* root rot tolerant avocado (*Persea americana*) rootstock to flooding and *Phytophthora cinnamomi*. BMC Plant Biol. 16: 205. doi: 10.1186/s12870-016-0893-2

Robin, C., Morel, O., Vettraino, A. M., Perlerou, C., Diamandis, S., and Vannini, A. (2006). Genetic variation in susceptibility to *Phytophthora cambivora* in European chestnut (*Castanea sativa*). Forest Ecol. Manag. 226, 199–207. doi: 10.1016/j.foreco.2006.01.035

Rosa, D. D., Campos, M. A., Targon, M. L. P. N., and Souza, A. A. (2007). *Phytophthora parasitica*

transcriptome, a new concept in the understanding of the citrus gummosis. Genet. Mol. Biol. 30, 997–1008. doi: 10.1590/S1415-47572007000500028

Shearer, B. L., and Tippett, J. T. (1989). Jarrah Dieback: the dynamics and management of *Phytophtora cinnamomi* in the Jarrah (*Eucalyptus marginata*) forest of south-western Australia. Department of Conservation and Land Management, Perth. Res. Bull. 3, 1–76.

Tainter, F. (1997). "Littleleaf disease," in *Compendium of Conifer Diseases* eds E.Hansen and K. J. Lewis (Saint Paul, MN), 4–6.

Tewoldemedhin, Y. T., Mazzola, M., Botha, W. J., Spies, C.F.J., and McLeod, A. (2011). Characterization of fungi (*Fusarium* and *Rhizoctonia*) and oomycetes (*Phytophthora* and *Pythium*) associated with apple orchards in South Africa. Eur. J. Plant Pathol. 130, 215–229. doi: 10.1007/s10658-011-9747-9
